# Molecular Therapy for Degenerative Disc Disease: Clues from Secretome Analysis of the Notochordal Cell-Rich Nucleus Pulposus

**DOI:** 10.1038/srep45623

**Published:** 2017-03-30

**Authors:** Ajay Matta, M. Zia Karim, David E. Isenman, W. Mark Erwin

**Affiliations:** 1 Krembil Research Institute, Toronto Western Hospital, Toronto, Ontario, Canada; 2 Department of Biochemistry, Faculty of Medicine, University of Toronto, Toronto, Ontario, Canada; 3 Department of Surgery, University of Toronto, Toronto, Ontario, Canada; 4 Division of Research, Canadian Memorial Chiropractic College, Canada

## Abstract

Degenerative disc disease (DDD) is associated with spinal pain often leading to long-term disability. However, the non-chondrodystrophic canine intervertebral disc is protected from the development of DDD, ostensibly due to its retention of notochordal cells (NC) in the nucleus pulposus (NP). In this study, we hypothesized that secretome analysis of the NC-rich NP will lead to the identification of key proteins that delay the onset of DDD. Using mass-spectrometry, we identified 303 proteins including components of TGFβ- and Wnt-signaling, anti-angiogeneic factors and proteins that inhibit axonal ingrowth in the bioactive fractions of serum free, notochordal cell derived conditioned medium (NCCM). Ingenuity Pathway Analysis revealed TGFβ1 and CTGF as major hubs in protein interaction networks. *In vitro* treatment with TGFβ1 and CTGF promoted the synthesis of healthy extra-cellular matrix proteins, increased cell proliferation and reduced cell death in human degenerative disc NP cells. A single intra-discal injection of recombinant TGFβ1 and CTGF proteins in a pre-clinical rat-tail disc injury model restored the NC and stem cell rich NP. In conclusion, we demonstrate the potential of TGFβ1 and CTGF to mitigate the progression of disc degeneration and the potential use of these molecules in a molecular therapy to treat the degenerative disc.

Degenerative disc disease (DDD) is a predominant contributor (~40%) to the genesis of spinal pain and is a major cause of disability worldwide, imposing enormous socio-economic burden and clinical costs to society^1^. The 2010 Global Burden of Disease Study estimated that low back pain is among the top 10 diseases and injuries that account for the highest number of disability-adjusted life years worldwide. It is estimated that approximately 25% of the population in United States of America suffers from lower back and neck pain at costs greater than $100 billion annually^2^. Current surgical treatments including fusion and disc replacement do not attempt to slow the degenerative process or promote repair^3^. In fact, a meta-analysis suggested that lumbar fusion might result in a higher prevalence of adjacent segment degeneration or disease than motion-preserving procedures^4^. Thus, there exists a need for the identification of novel, effective therapeutic agents that can be clinically translated as minimally invasive regenerative therapies for disc repair. Several approaches including gene therapy, growth factors, stem cells and tissue engineering have been proposed for the treatment of disc degeneration^5^,^6^,^7^,^8^,^9^,^10^. Growth factors including insulin-like growth factor-1 (IGF-1), fibroblast growth factor-2 (FGF-2) and morphogens such as bone morphogenetic protein-2 (BMP-2), osteogenic protein-1(OP-1) and growth differentiation factor-5 (GDF-5) have been investigated for their potential to stimulate extracellular matrix (ECM) synthesis in intervertebral disc (IVD) cells^6^. However, the use of BMP-2 for the treatment of DDD in humans is limited by its osteo-inductive characteristics^11^. Similarly, the injection of rhBMP-7/OP-1 resulted in exogenous bone formation with no evidence of disc regeneration in the beagle IVD - NP^12^. Moreover, administration of anti-inflammatory agents including interleukin 1 receptor antagonist (IL-1Ra) and synthetic inhibitors targeting tumor necrosis factor α (TNFα) or nuclear factor kappa B (NFκB) have shown limited efficacy in stimulating the anabolic response^13^,^14^. Therefore, the identification of novel biologically active agents that can restore the homeostatic microenvironment in a degenerative disc would revolutionize the treatment of DDD.

The intervertebral disc (IVD) is composed of a central nucleus pulposus (NP) surrounded by the concentric annulus fibrosus and attached to the adjacent vertebrae by thin cartilaginous end plates^15^,^16^. The NP plays an important role in maintaining the biomechanical properties of the spine such that in youth the healthy NP is rich in large, vacuolated notochordal cells (NCs) and possesses a proteoglycan rich ECM. However, NCs observed during childhood are gradually replaced by small chondrocyte-like cells (CLCs) by early adolescence in humans, a NP cellular phenotype often associated with disc degeneration^15^,^16^. The degenerative disc NP is a catabolic phenotype representing a failure of homeostatic regulation of the microenvironment with the increased expression of pro-inflammatory cytokines and matrix metalloproteinases (MMPs) leading to progressive cell death, loss of proteoglycan content and development of an inferior, fibrocartilaginous extra-cellular matrix (ECM). This affects IVD structural integrity, compromising its biomechanical properties often leading to pain, neurological compromise and disability^15^,^16^,^17^,^18^,^19^. With respect to the development of DDD, a temporal relationship has been suggested between the loss of NCs and the progression of DDD^15^,^16^,^17^.

Unlike humans, non-chondrodystrophic (NCD) canines, porcine and rodents preserve large, vacuolated NCs within their NPs and are relatively resistant to DDD, until much later in their lives^19^. In our previous studies, we reported that conditioned medium collected from alginate cultures of notochordal cells (NCs) derived from NCD-canines reduced cell death and stimulated collagen 2 synthesis in bovine NP cells^20^,^21^. Similarly, other studies have demonstrated increased proteoglycan synthesis in NP cells treated with notochordal cell derived conditioned medium (NCCM) *in vitro*^22^. A recent study also demonstrated the anti-angiogeneic effects of NCCM *in vitro*^23^. These studies provide compelling evidence that NCs secrete proteins, which may confer anabolic characteristics, maintain a homeostatic microenvironment in NP and resists DDD. We hypothesized that secretome analysis of NC-rich NPs will give us insight into the protein networks regulating the development of DDD and may be exploited for regenerating the degenerative discs in humans. In this study, we identified the proteins in serum free NCCM using mass spectrometry and, improved and characterized a pre-clinical rodent disc - injury model of DDD. We used Ingenuity Pathway Analysis (IPA) to identify protein networks that might be of significance in the homeostatic regulation of the healthy disc NP. Next we evaluated the regenerative and anti-inflammatory potential of connective tissue growth factor (CTGF) and transforming growth factor beta 1 (TGFβ1), identified as major hub proteins in network analysis, using *in vitro* assays and an *in vivo* pre-clinical rodent disc - injury model of DDD.

## Results

### Evaluation of a pre-clinical rat-tail disc injury model of DDD

**Figure 1:**
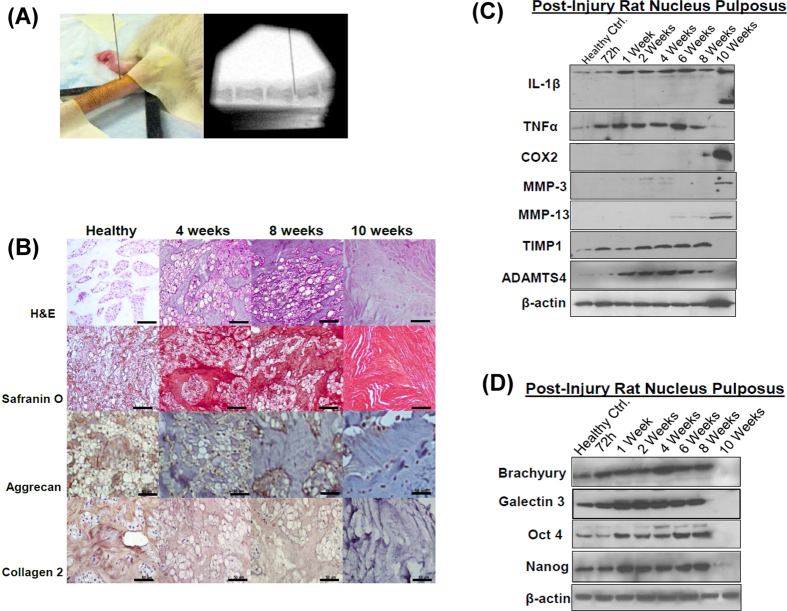
Needle puncture injury in rat-tail disc leads to the development of fibrocartilaginous matrix and loss of notochordal (NC) and stem cells in nucleus pulposus (NP). (**A**) Representative fluoroscopic image-guided needle puncture injury in rat-tail disc NP. (**B**) Histological analysis demonstrating representative hematoxylin and eosin (H&E) and Safranin O staining images showing development of a fibrocartilaginous matrix over a period of 10 weeks p.i. in rat NP. Both H&E and Safranin O staining was done in duplicates on IVD sections obtained from all the animals in each group. Immunohistochemistry showing the loss of the ECM proteins, aggrecan and collagen 2 in time dependent manner (healthy to 10 weeks p.i., Scale bar 50μ, n = 2 per group). (**C**) Representative Western blot panels showing alterations in the expression of pro-inflammatory cytokines (IL-1β and TNFα), inflammation mediator, Cox2 and ECM proteins (MMP-3, MMP-13, TIMP1 and ADAMTS4) in a time dependent manner in post-injury rat NP tissue lysates. (**D**) Representative Western blot panels showing loss of NC markers (brachyury, galectin 3) and stem cell markers (Oct4, Nanog) in NPs obtained from rat-tail injured discs over a period of 10 weeks. β-actin was used a loading control in Western blots. All Western blots were run under the same experimental conditions as described in Materials and Methods.

We characterized a pre-clinical rodent model of DDD to establish a platform for the evaluation of therapeutic agents. Using fluoroscopic image guidance, we performed needle puncture injuries using a 27-gauge needle in the caudal (tail) discs of 12 week-old, healthy Wistar rats (n = 21, 4 discs per animal). Alterations in the composition of the ECM and the cellular phenotype were determined in a time dependent manner (72 hrs–10 weeks, Fig. 1A–D). Histological analysis revealed a gradual loss of NCs, increased Safranin-O staining intensity and reduced expression of aggrecan and collagen 2 by the end of 10 weeks post injury (p.i.) in comparison to uninjured, healthy control IVD-NP obtained from age matched healthy controls (Fig. 1B, Supplementary Fig. 1A). Of note, needle puncture injury increased the expression of the pro-inflammatory cytokines, TNFα within 72 hrs and interleukin-1β (IL-1β) as early as 1 week p.i. (Fig. 1C, Supplementary Fig. 1B). However, the active form of IL-1β (~17 kDa) was not observed until between 8–10 weeks, coincident with an increased expression of the inflammatory mediator, cyclooxygenase 2 (COX2) and the ECM degrading enzymes, matrix metalloproteinases-3 (MMP-3) and MMP-13 as compared to no treatment controls (NTCs, i.e. tissue lysates obtained from uninjured IVD-NPs, Fig. 1C, Supplementary Fig. 1B). Interestingly, the loss of tissue inhibitor of metalloproteinases 1 (TIMP1), a natural inhibitor of MMPs was also observed at the end of 10 weeks p.i. coincident with the onset of increased levels of the MMPs (Fig. 1C, Supplementary Fig. 1B). We also observed increased expression of A disintegrin-like and metalloprotease with thrombospondin type 1 motif 4 (ADAMTS4), one of the major enzymes responsible for aggrecan degradation (Fig. 1C, Supplementary Fig. 1B). In addition to ECM changes, we also determined the expression of notochordal and stem cell markers in healthy and injured IVD-NP lysates. Our western blotting results demonstrated the loss of the NC markers (brachyury and galectin 3) and stem cell markers (Oct4 and Nanog) in the injured disc NPs, 10 weeks p.i. (Fig. 1D, Supplementary Fig. 1C). These findings clearly demonstrated a shift in the NP *milieu* from a healthy, homeostatically regulated environment to a pro-inflammatory, catabolic state with loss of both NCs and stem cells, similar to the human degenerative disc NP. Comparative analysis of the histological characteristics of human degenerative disc NPs with bovine, NCD - canine and Wistar rat IVDs is described in Supplementary Data (Supplementary Fig. 2A). Negative control tissue sections (rat healthy/mongrel IVD – NP) wherein the primary antibody was replaced by rabbit or goat isotype IgG controls revealed no immunostaining thereby demonstrating the specificity of the antibodies (Supplementary Fig. 2B).

### Intra-discal injection of NCCM promotes regeneration of the degenerative disc nucleus pulpous in a pre-clinical rodent model of DDD

**Figure 2:**
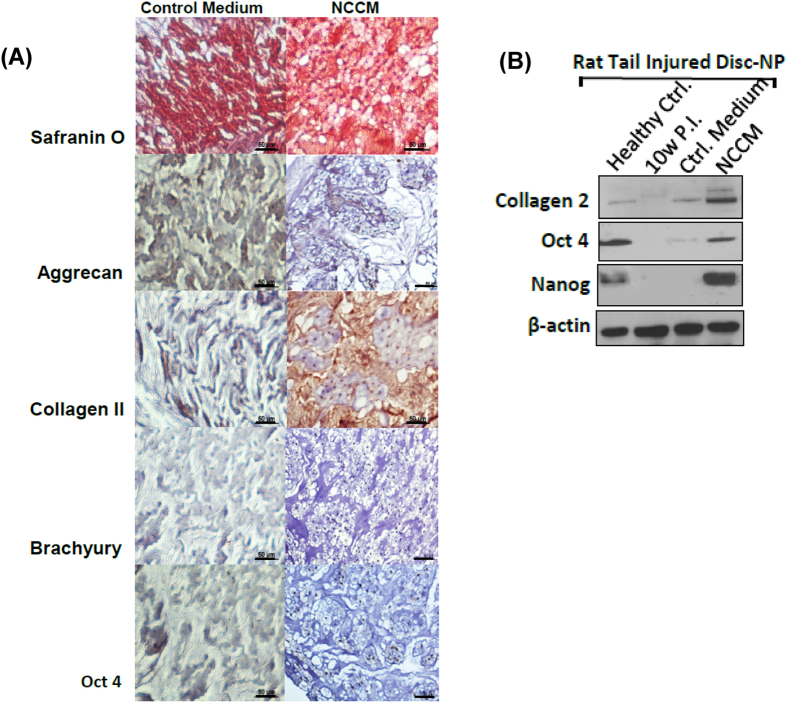
NCCM confers anabolic and anti-catabolic characteristics to degenerating NP in rat-tail injured IVD - NP. (**A**) Representative Safranin O staining sections showing fibro-cartilaginous matrix and small NP cells in rat tail injured disc NPs injected with control medium as compared to the injured disc NPs injected with serum free NCCM showing proteoglycan-rich ECM with large notochordal cells (n = 12 tissue sections/group). Representative panel showing immunohistochemistry of aggrecan, collagen 2, brachyury and Oct4 in paraffin embedded sections of rat-tail injured discs treated with protein free Hybridoma medium used as control or NCCM (Scale bar 50μ). Immunohistochemistry for all the proteins was performed in duplicates in at least 3 different IVD sections obtained from different animals from each group used in this study. (**B**) Representative Western blot panels showing expression of collagen 2 and the stem cell markers Oct4 and Nanog in tissue lysates obtained from healthy controls, rat-tail injured disc NPs left untreated, injected with control medium or NCCM.

We injected concentrated, serum free NCCM or control medium (~8 μL/disc) into the injured (4-week p.i.) rat-tail disc NPs (n = 6 animals/group, 4 discs per animal) using a specially designed, 32-gauge needle with a 35 degree bevel under fluoroscopic guidance. Ten weeks p.i., histological analysis revealed NC-rich NPs with moderate Safranin-O staining in NCCM injected rat-tail injured discs (Fig. 2A). In contrast, rat-tail injured discs injected with control medium showed low cellularity and displayed a fibrocartilaginous matrix, with intense Safranin-O staining (Fig. 2A). Immunohistochemistry and western blotting revealed that discs injected with NCCM displayed restoration of aggrecan, collagen 2, brachyury, Oct4 and Nanog in contrast to the sham/control medium injected discs (Fig. 2A,B, Supplementary Fig. 2C). However, protein extraction using RIPA lysis buffer is a potential limitation for estimation of Collagen2 expression in tissues using Western blotting. Nonetheless, these results validated our hypothesis and demonstrated that soluble factors within NCCM have therapeutic potential for a degenerative IVD - NP.

### Identification of soluble factors in NCCM using mass spectrometry

**Figure 3:**
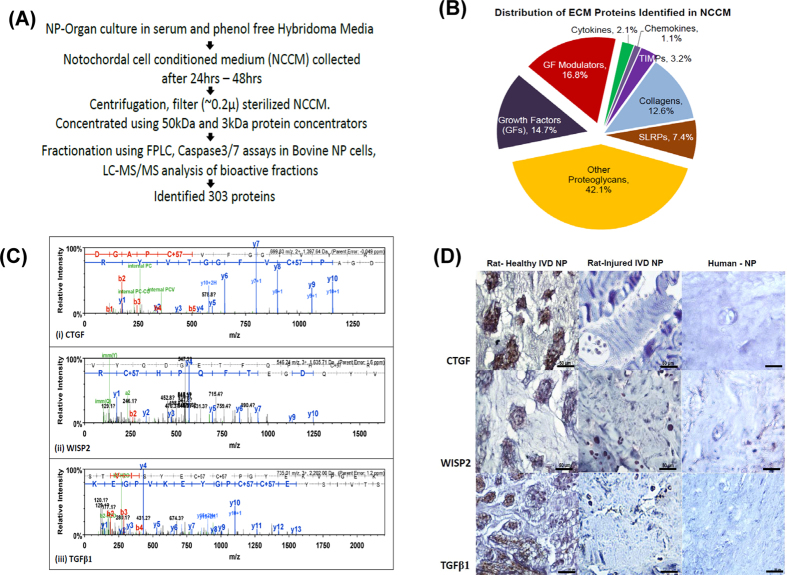
Analysis of NCCM. (**A**) Schematic representation of the methodology for identification of proteins in NCCM using mass - spectrometry. (**B**) Pie-chart showing distribution of ECM proteins identified in NCCM. (**C**) Peptide signature peaks for CTGF, WISP2 and TGFβ1 observed in mass – spectrometry analysis of NCCM. (**D**) Immunohistochemistry showing expression of CTGF, WISP-2 and TGFβ1 in paraffin-embedded sections of rat NP (healthy and injured discs) and human degenerative disc NP (Scale bar 50μ).

Serum-free NCCM was concentrated sequentially using 50 kDa and 3 kDa filters, followed by fractionation using size exclusion chromatography (Fig. 3A). Of the 30 fractions collected, only five of these fractions (50PF4, 50PF5, 50PF6, 3PF4 and 3PF5) suppressed etoposide - induced caspase-3/7 activity in bovine NP cells (Supplementary Fig. 3A–C). Using mass-spectrometry analysis, we identified 303 non-redundant proteins in these bioactive fractions of NCCM, of which 31% proteins have been reported within the ECM (Fig. 3B, Table 1, Supplementary Table S1). We identified several growth factors including transforming growth factor beta 1 (TGFβ1), connective tissue growth factor (CTGF), Wnt-induced soluble protein 2 (WISP2), insulin-like growth factor binding protein 7, angiopoietin-like 7 and their modulators, including chordin, sclerostin and cartilage intermediate layer protein (CILP, Fig. 3C, Table 1). For a complete list of proteins see Supplementary Table S1. Immunohistochemical analysis demonstrated moderate to strong immunostaining of CTGF, Wnt–inducible soluble protein 2 (WISP2) and TGFβ1 in NC cells and ECM in healthy rat-tail disc NPs. However, no detectable expression of CTGF, WISP2 or TGFβ1 was observed in the ECM of degenerative needle puncture injured rat-tail or human disc NPs (Fig. 3D). These findings suggested an association between the loss of CTGF, WISP2 and TGFβ1 with the development of DDD.

### Network Analysis of Proteins Identified in Secretome Analysis

**Figure 4:**
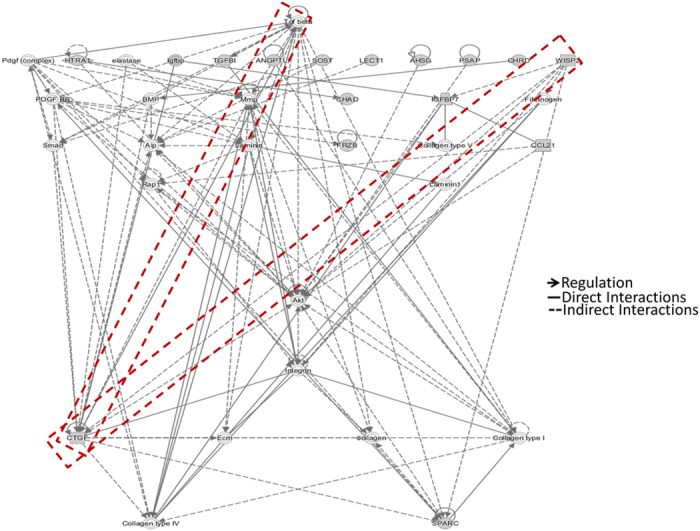
Ingenuity Pathway Analysis (IPA). IPA was performed using 94 ECM proteins identified in our secretome analysis to determine the significant protein networks. Hierarchical model showing TGFβ1 as a major hub protein, interacting with or regulating expression of several other proteins in the network. Bold arrows show direct regulation, bold lines show direct interactions while dotted lines represent indirect interactions among proteins shown in the network.

Using Ingenuity Pathway Analysis (IPA) software, we analyzed the networks inherent to the healthy, notochordal cell rich NP. Among the major signaling pathways, TGFβ, Wnt/β-catenin, axonal guidance, nitric oxide synthase (eNOS), leucocyte extravasation signaling and inflammation associated matrix degradation emerged as significant pathways (p < 0.05, Supplementary Fig. 4A–E). In a hierarchical model, TGFβ1 emerged as the top protein hub interacting or regulating the expression of several other proteins including CTGF, platelet-derived growth factor (PDGF), secreted protein acidic and rich in cysteine (SPARC), MMPs and collagens identified in this study (Fig. 4). In another model, CTGF appeared as a central hub protein interacting with BMPs, TGFβ1, Smads, WISP2, several ECM proteins (aggrecan, collagens, decorin, HAPLN1, fibulin1) and kinases (Supplementary Fig. 4B).

### Evaluation of the anabolic effects of CTGF, WISP2 and TGFβ1 on NP cells

**Figure 5:**
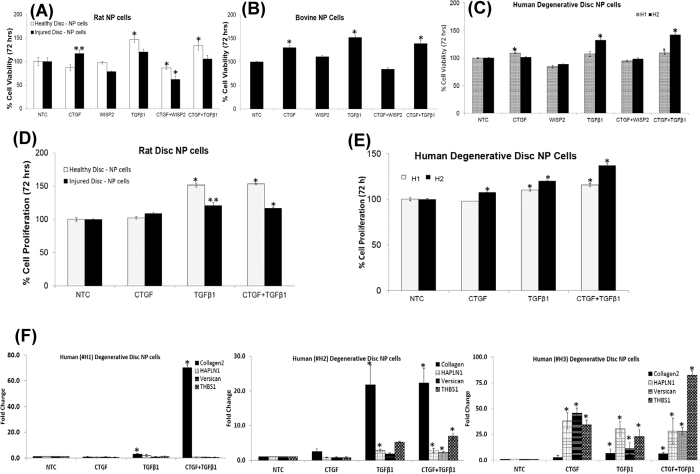
Anabolic effects of CTGF, WISP2 and TGFβ1 in an *in vitro* model of DDD. Effect of CTGF, WISP2 and TGFβ1 treatment alone or in combination on cell viability (72 hrs) as determined using MTT assays in NP cells obtained from (**A**) rat tail (healthy/injured) discs **p = 0.049, *p ≤ 0.02), (**B**) bovine degenerative disc NPs, *p < 0.01 and (**C**) human (H1, H2) degenerative disc NPs (*p < 0.005). Each bar represents mean ± S.D. of 3 independent experiments done in triplicates (n = 9). Cell proliferation assays (72 hrs) in (**D**) rat NP cells (healthy/injured discs), *p < 0.001, **p < 0.01 and (**E**) human (H1, H2) degenerative disc NPs treated with CTGF and TGFβ1 alone or in combination as determined using colorimetric anti-BrdU – ELISA, *p < 0.001. The p-values were determined using paired Student’s t-test, for treatment with CTGF, WISP2 or TGFβ1 alone or in combination with respect to no treatment control (NTC). (**F**) Histograms showing alterations in expression of collagen 2, HAPLN1, versican and thrombospondin1 (Thbs1) on treatment of human degenerative disc NP cells (H1, H2, H3) with CTGF and TGFβ1 as revealed by real time PCR analysis. Each bar in the histogram represents the mean ± S.D. of 3 independent experiments done in duplicates (n = 6, *p < 0.001).

We treated rat-tail disc NP cells with recombinant human (rh) CTGF, WISP2 or TGFβ1 proteins in order to evaluate their effect upon cell viability in a dose and time dependent manner (5 ng/mL–100 ng/mL, 24 hr–96 hrs, Supplementary Fig. 5A–D). Treatment with CTGF (100 ng/ml) or TGFβ1 (10 ng/ml) alone or in combination increased the viability of NP cells derived from healthy rat-tail discs and degenerative discs (rat-tail injured discs, bovine and human) in 48 hrs–72 hrs (Fig. 5A–C, Supplementary Fig. 6A–C). Treatment with a combination of CTGF (100 ng/ml) and TGFβ1 (10 ng/ml) increased cell viability by ≥35% within 72 hrs in human degenerated disc NP cells (Fig. 5C, Supplementary Fig. 6C). However, treatment with WISP2 showed no significant differences in cell viability of NP cells derived from rat, bovine and human discs (Fig. 5A–C). This was further confirmed with cell proliferation assays using bromodeoxyuridine (BrdU) incorporation. We observed a significant increase in DNA synthesis in rat-tail (healthy/injured) and human degenerated disc NP cells upon treatment with TGFβ1 (10 ng/ml) alone or in combination with CTGF (100 ng/ml) within 72 hrs (Fig. 5D,E). We also observed increased mRNA levels of important ECM proteins including collagen type 2, hyaluronan and proteoglycan link protein 1 (HAPLN1), versican and thrombospondin1 (THBS1) in human degenerative disc NP cells upon treatment with a combination of CTGF and TGFβ1 (Fig. 5F). Western blotting verified an increase in collagen 2 expression on treatment with a combination of CTGF and TGFβ1 within 24 hrs (Supplementary Fig. 7A and B), supporting their anabolic roles on human degenerated disc NP cells.

### Evaluation of anti-catabolic effects of CTGF, WISP2 and TGFβ1 on NP cells

**Figure 6:**
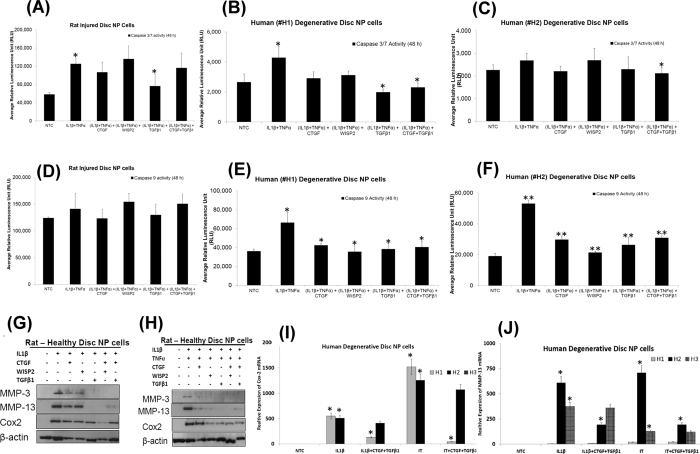
Anti-catabolic effects of CTGF, WISP2 and TGFβ1 in an *in vitro* model of DDD. Histograms showing caspase 3/7 activity in NP cells derived from (**A**) rat injured IVD (*p = 0.008), (**B**,**C**) human degenerative disc NP treated with pro-inflammatory cytokines, IL-1β and TNFα alone or in presence of CTGF, WISP2 and TGFβ1 (*p < 0.05). Histograms showing caspase 9 activity in NP cells (**D**) rat injured IVD, (**E**,**F**) human degenerative disc NP cells treated with pro-inflammatory cytokines, IL-1β and TNFα alone or in presence of CTGF, WISP2 and TGFβ1(*p < 0.05, **p < 0.005). Each bar in the caspase assays is showing mean ± S.D. of 2 independent experiments done in quadruplets (n = 8). The p-values were determined using paired Student’s t-test. For the combination of IL-1β and TNFα are with respect to no treatment control (NTC), while p-values for the groups containing growth factors (CTGF, WISP2 or TGFβ1) are with respect to the group containing combination of IL-1β and TNFα only. Representative Western blot panels showing expression of MMP-3, MMP-13 and Cox2 in rat healthy IVD NP cells treated with (**G**) IL-1β alone, (**H**) IL-1β and TNFα in combination, and in the presence of CTGF, WISP2 or TGFβ1. All Western blots were run under the same experimental conditions as described in Materials and Methods. Histograms showing decreased expression of (**I**) Cox2, (**J**) MMP-13 mRNA levels in human degenerative disc NP cells treated with IL-1β and TNFα in presence of CTGF and TGFβ1 in comparison to IL-1β and TNFα only treatments. Each bar in the histogram represents mean ± S.D. of 3 independent experiments done in duplicates (n = 6, *p < 0.001).

Human and rat NP cells were treated with pro-inflammatory cytokines, IL-1β (10 ng/ml) alone or a combination of IL-1β (10 ng/ml) and TNFα (50 ng/ml) in the presence of CTGF (100 ng/ml), WISP2 (100 ng/ml) and TGFβ1 (10 ng/ml) alone or in combination for 24–48 hrs. Treatment with IL-1β alone or a combination of IL-1β and TNFα induced apoptosis as revealed by increased caspase-3/7 activity in NP cells (Fig. 6A–C). However, a significant decrease in inflammation–induced caspase-3/7 activity was observed in degenerative disc NP cells (rat/human) in the presence of TGFβ1 alone or combined with CTGF (Fig. 6A–C). A significant reduction in IL-1β and TNFα induced caspase-9 activity was also observed in the presence of CTGF, WISP2 or TGFβ1 in human degenerative disc NP cells (Fig. 6E,F). Treatment with CTGF, WISP2 or TGFβ1 reduced expression of MMP-3, MMP-13 and Cox2 proteins in rat NP cells treated with IL-1β and TNFα (Fig. 6G,H, Supplementary Fig. 8, A and B). Treatment of human degenerative NP cells with the pro-inflammatory cytokines, IL-1β and TNFα, induced expression of Cox2 (H1 and H2) and MMP13 in all 3 cases (H1, H2 and H3) as compared to their respective no treatment controls (NTC, Fig. 6I,J). However, the addition of CTGF and TGFβ1 reduced Cox2 and MMP-13 mRNA levels induced by IL-1β and TNFα in human degenerative discs NP cells demonstrating their anti-catabolic effects (Fig. 6I,J).

### Intra-discal injection of CTGF and TGFβ1 mediates progression of degenerative disc disease in a pre-clinical rodent model

**Figure 7:**
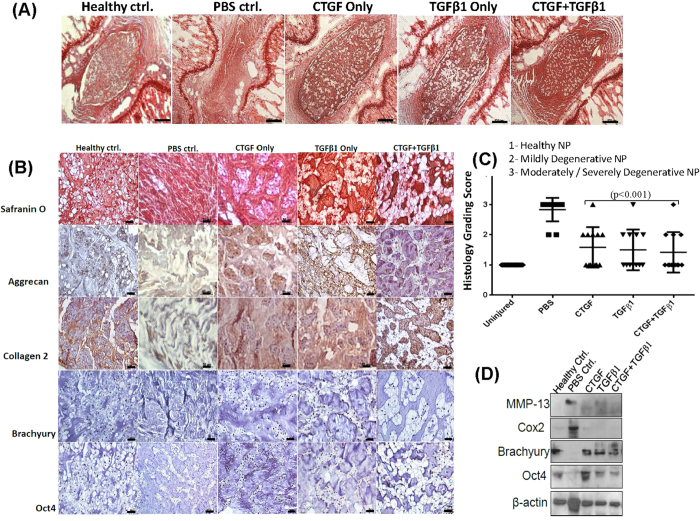
Evaluation of the therapeutic potential of CTGF and TGFβ1 in a pre-clinical *in vivo* rodent disc injury model of DDD. (**A**) Representative Safranin-O staining photomicrographs representing uninjured, healthy IVD and injured rat IVDs injected with phosphate - buffered saline (PBS, used as a control), CTGF, TGFβ1 or a combination of CTGF and TGFβ1 (Scale bar 500μ, n = 12 tissue sections/group). (**B**) Representative Safranin-O and immunohistochemical staining of ECM proteins, aggrecan and collagen 2 in paraffin embedded sections of rat tail injured IVD - NPs treated with phosphate - buffered saline (PBS, used as a control), CTGF, TGFβ1 or a combination of CTGF and TGFβ1 (Scale bar 50μ). Immunohistochemistry for all the proteins was performed in duplicates in atleast 3 different IVD sections obtained from different animals from each group. (**C**) Scatter plots showing histological grading scores (mean ± S.D.) of rat healthy tail disc-NPs and injured tail disc-NPs following treatment with PBS, CTGF, TGFβ1 and a combination of CTGF and TGFβ1 (**p < 0.001). The p-values were determined for treatment with CTGF, TGFβ1 alone or in combination with respect to PBS. (**D**) Representative Western blot panels showing decreased expression of MMP-13 and Cox2, and restoration of the NC marker, brachyury and stem cell marker, Oct4 in NP tissue lysates (pooled) obtained from rat tail injured discs treated with CTGF, TGFβ1 or a combination of CTGF and TGFβ1.

To test the potential of CTGF and TGFβ1 to mediate progression of DDD in a pre-clinical rodent model, we performed image guided rat-tail disc injuries (n = 24) in two independent experiments. Four weeks following injury, the animals were randomized into four groups (n = 6 animals/group) and an intra-discal injection (~8 μL) of CTGF (100 ng/mL i.e. 0.80 ng/disc), TGFβ1 (10 ng/mL, i.e. 0.08 ng/disc), a combination of CTGF (100 ng/mL) and TGFβ1 (10 ng/mL) or phosphate buffered saline (PBS, 1X, pH = 7.2) as a vehicle control was administered in each injured disc NPs (4 discs per animal). Histological analysis of discs injected with PBS (1X, pH = 7.2) showed a fibrocartilaginous, acellular matrix with intense Safranin-O staining within the NP (Fig. 7A–C). However, injured rat-tail discs treated with CTGF, TGFβ1 alone or in combination, demonstrated a healthy disc, rich in NCs, 10 weeks p.i. (Fig. 7A–C). Immunohistochemical analysis confirmed the restoration of a healthy NP on treatment with rh CTGF or TGFβ1, demonstrating strong expression of aggrecan, collagen 2, Brachyury and Oct4 as compared to injured disc NPs injected with PBS (1X, pH = 7.2) used as sham controls (Fig. 7B). Western blotting validated the restoration of Brachyury and Oct4 expression in rat-tail injured disc NPs following treatment with rh-CTGF and TGFβ1 proteins (Fig. 7D, Supplementary Fig. 9A). Further, treatment with CTGF and TGFβ1 also suppressed levels of the ECM degrading enzyme, MMP-13 and inflammation mediator, cyclooxygenase-2 (Cox2) in injured disc NPs (Fig. 7C, Supplementary Fig. 9A).

## Discussion

Degenerative disc disease (DDD) is associated with the progressive loss of notochordal cells (NCs) and the development of an inferior NP matrix compromising the structural integrity and biomechanical properties of the spine^15^,^16^,^17^,^18^,^19^. In this study, we characterized a pre-clinical rodent disc injury model that mimics progressive disc degeneration, similar to that observed in humans. Our *in vitro* and pre-clinical rodent model emphasized the role of inflammation in deterioration of the NP - ECM and cell death during progressive disc degeneration. Further, we have identified the key factors secreted by NCD-canine notochordal cells responsible for maintaining a healthy, hydrophilic, proteoglycan rich IVD NP that resists DDD. Our secretome analysis of the NC-rich nucleus pulposus revealed components of TGFβ- and Wnt-signaling, anti-angiogeneic factors (semaphorins, nidogen), several proteogylcans including aggrecan, small leucine rich proteoglycans (SLRPs) and collagens in serum free NCCM. Both TGFβ1 and CTGF emerged as the major protein hubs regulating the expression of several ECM proteins and interacting with other growth factors in protein network analysis. These protein networks are important for maintenance of a healthy disc NP and might be involved in resisting the development of DDD.

Both TGFβ1 and CTGF play a critical role in the development of the IVD and cartilage in the embryonic stage as well as in post-natal development of the spine^24^,^25^. TGFβ1 belongs to the transforming growth factor beta (TGFβ) superfamily of proteins known to play an important role in cartilage and bone development^26^. CTGF is an important constituent of the intervertebral disc microenvironment and interacts with several growth factors and matrix proteins including integrins and heparan sulfate proteoglycans^27^,^28^,^29^,^30^,^31^. CTGF and WISP2 (CCN5) belong to the connective tissue growth factor/cysteine-rich 61/nephroblastoma overexpressed (CCN) - family of matricellular proteins that possess an amino-terminal secretory peptide followed by four conserved domains with sequence homologies to insulin-like growth factor-binding proteins, von Willebrand factor C (VWC) domain, thrombospondin type 1 repeat (TSR) and a carboxy-terminal (CT) domain that contains a cysteine-knot motif^27^. Interestingly, the von Willebrand factor C (VWC) domain present in CTGF mediates physical interactions with members of the TGFβ-superfamily, including bone morphogenetic proteins (BMPs) and other TGFβ ligands^27^,^28^,^29^,^30^,^31^. In contrast, CCN5 negatively regulates TGFβ signaling by suppressing the expression of TGFβRII. Knocking down expression of CCN5 in breast cancer cells (MCF-7) resulted in deregulation of several components of the TGFβ - signaling pathway including its downstream effector SMAD signaling proteins^32^.

Our data showed the loss of TGFβ1, CTGF and WISP2 expression in degenerative disc NPs implicating their association with the loss of NCs. Notably, treatment with a combination of TGFβ1 and CTGF restored a healthy, cellular NP rich in NC and stem cells as compared to sham control discs in our pre-clinical models. In addition, our results demonstrated that treatment with TGFβ1 and CTGF increased cell viability, DNA synthesis and expression of healthy ECM genes in NP cells derived from degenerative human discs. The mitigation of inflammation induced caspase-3/7 activity, Cox2, MMP-3 and MMP-13 expression levels by recombinant TGFβ1 and CTGF demonstrated their synergistic action. CTGF has been shown to suppress IL-1β - induced mRNA levels of MMP-3, ADAMTS5, syndecan-4 and prolyl hydroxylase 3 by binding to cell surface integrin receptors α_v_β_3_ and α_5_β_1_ in healthy NP cells^33^. These finding clearly suggest the potential overlap in the cell signaling activated downstream of TGFβ1 and CTGF regulating their anti-inflammatory and anti-catabolic activities in NP cells. However, our study is limited to studying the changes in the cellular and biochemical composition of NP in response to inflammation *in vitro* and disc injury *in vivo* but lacks determination of changes in disc height, if any. Further investigation needs to done in animal models to determine if such treatments have the capacity to retain or gain increased disc height in addition to biochemical changes in response to these treatments.

Among other proteins, we identified CD109, COMP, CILP, decorin, chordin and sclerostin proteins that are known activators or modulators of TGFβ-Smad dependent signaling. Soluble CD109 can bind to all 3 isoforms of TGFβ-1/2/3, thus interfering with the activation of TGFβ dependent signaling^34^. In fact, membrane bound CD109 acts as a co-receptor for TGFβ1 and results in internalization and degradation of TGFβ receptors via binding to Smad7^35^,^36^. Further, CILP inhibits transcriptional activation of matrix genes in NP cells by binding to TGFβ1 and inhibiting the downstream phosphorylation of Smads, thereby promoting disc degeneration^37^. In addition, small leucine rich proteoglycans (SLRPs) like decorin sequester multiple growth factors including TGFβ1 and directly antagonizes the function of EGFR and IGF-IR^38^. In contrast, COMP activates TGF-β dependent transcriptional activity^39^. Ishida *et al*.^39^, proposed that COMP may serve as an “instructive matrix” component *in vivo* slowing the diffusion of the growth factors and promoting their biological activity. Similarly, sclerostin binds to BMP-6 and BMP-7 while chordin binds to BMP-2 and BMP-4 preventing their interaction with BMP receptors^40^. Taken together, these studies suggest the presence of a delicate balance in TGFβ- signaling in NC - rich NPs that is well regulated and responsible for maintaining a healthy, proteoglycan rich NP.

In addition, sclerostin and soluble frizzled-related protein 1 (sFRP1) identified in NCCM are known antagonists of Wnt-signaling pathway. The secreted frizzled-related proteins (sFRP) bind to Wnt ligands, whereas the sclerostin binds to components of the Wnt receptor^41^. We also identified LRP1, LRP2 and VLDLR, which act as receptors for activation of Wnt-signaling. Hiyama *et al*.^42^, reported that the activation of Wnt signaling upregulates the pro-inflammatory cytokine TNF-α leading to disc degeneration. Thus blocking Wnt signaling might protect nucleus pulposus cells against degeneration^43^. Further, Iwata *et al*.^44^, demonstrated that Wnt/β-catenin signaling could enhance Runx2 expression in NPs resulting in IVD calcification. Thus, cross talk between TGFβ- and Wnt-signaling pathways might play an important role in the development and progression of DDD. However, these findings require further investigation in order to understand their impact on the development and progression of DDD.

Another class of soluble factors identified in NCCM included proteins that prevent the ingrowth of blood vessels and neurites into the NP, a hallmark of DDD. Angiopoeitin like 7 (ANGPTL7) has been proposed as an anti-angiogeneic protein abundantly expressed in keratocytes and plays a major role in maintaining corneal avascularity and transparency^45^. Semaphorin 3E identified in NCCM belongs to class 3 semaphorin family of proteins, known as potent inhibitors of both pathological nerve innervation and vascular proliferation^46^,^47^. Another member of the family, Semaphorin 3A is highly expressed by healthy disc cells, primarily localized to the outer annulus fibrosus, but decreases significantly in the degenerative disc nucleus pulposus^46^,^47^. Further, low expression of semaphorin 3A showed a significant correlation with overexpression of endothelial cell marker, CD31 and PGP9.5, marker for neurons^47^. However, the role of ANGPTL7 and Semaphorin 3E in disc degeneration is currently unknown and requires further investigations.

## Conclusions

**Figure 8:**
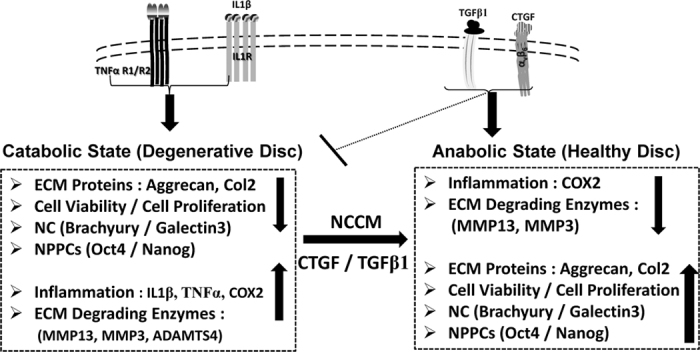
Proposed model demonstrating the mechanism of progressive disc degeneration in presence of pro-inflammatory cytokines (IL-1β and TNFα) and effect of intervention by novel, potential therapeutic agents (CTGF/TGFβ1) for regeneration of the IVD - NP.

In conclusion, we have identified the soluble proteins present within NCCM that have the ability to maintain a healthy, proteoglycan rich nucleus pulpous and delay DDD. We have further identified the proteins networks important for homeostatic regulation of the healthy IVD - NP. Our findings suggest that the loss of TGFβ1, CTGF and WISP2 is associated with the progression of DDD. Both TGFβ1 and CTGF represent pivotal protein hubs involved in cellular signaling and ECM regulation of the healthy IVD NP. We further demonstrated the potential utility of a combination of TGFβ1 and CTGF as novel, minimally invasive molecular therapeutic agents for the biological treatment of degenerative disc disease (Fig. 8).

## Materials and Methods

### Generation of serum free notochordal cell derived conditioned medium (NCCM)

Notochordal cell derived conditioned medium (NCCM) was collected from notcohordal cell- rich nucleus pulpous (NP) obtained from IVDs of non-chondrodystrophic (NCD) canines as described earlier^20^,^21^. All animals (n = 12) were obtained in collaboration with a licensed animal facility and all experimental protocols were approved by the Animal Care Committee, Research Animal care policy and Ethics Approval Board of University Health Networks, Toronto, Ontario, Canada. All experimental procedures were performed in accordance with the guidelines approved by Animal Care Committee and Ethics Approval Board of University Health Networks, Toronto, Ontario, Canada. All non-chondrodystrophic canines were 8 to 14 months of age and had failed at adoption or were to be euthanized for other purposes. Deep sedation was achieved using a combination of Acepromazine (10 mg/mL, Atravet-Aerst Pharmaceuticals St. Laurent, Quebec, Canada) mixed with Xylazine 100 mg/mL (Xylomax-Bimeda-NHC Animal Health, Broomhill Road, Tallaght, Dublin, Ireland) at a combined dose of 1 mL/15 Kg body weight. Once deep sedation had occurred, euthanasia was accomplished using intravenous sodium pentobarbital (CDMV) (St. Hyacinthe, Quebec, Canada at a dose of 30 mL/kg body weight. Within 2 hrs of euthanization, the lumbar spines were removed and nuclei pulposi were isolated under aseptic conditions^20^,^21^. Nuclei pulposi were washed with phosphate buffered saline (PBS, pH = 7.2) and three NPs were placed within tissue culture inserts with 40μm-filters in serum free CD Hybridoma media (protein and phenol red free, Cat No #11279-023, Life Technologies, USA) containing 100 units penicillin/streptomycin in 6 well plates under hypoxic conditions (3.5% O_2_, and 5% CO_2_, NuAire incubators) at 37 °C. The conditioned medium referred hereafter as NCCM was collected after 24 hrs – 48 hrs, centrifuged at 8000 rpm for 30 minutes, filtered through 0.2μm syringe-tip filters and stored in −80 °C until further use.

### Identification of bioactive fractions of NCCM

Notochordal cell condition medium (NCCM) was concentrated sequentially using 50 kDa and 3 kDa spin-ultrafiltration protein concentrators (EMD Millipore, MA, USA) following manufacturer’s instructions. The respective concentrated NCCM samples were further fractionated by size - exclusion on a Superose 12 HR 10/30 fast protein liquid chromatography (FPLC) column (Pharmacia) in running buffer (10 mM sodium phosphate, 150 mM NaCl, 1 mM EDTA, pH = 7.4). Thirty fractions (~1 ml) were collected, measured for protein concentration by absorbance at 280 nm and stored at −80 °C until further use. Consecutive protein-containing fractions were pooled pairwise and evaluated for their effect on etoposide (cytotoxic drug) induced caspase-3/7 activity in bovine tail disc NP cells as described below. Bioactive fractions were defined as protein fractions showing a decrease in caspase-3/7 activity in bovine NP cells on treatment with etoposide. These bioactive fractions were later analyzed for identification of proteins using mass-spectrometry.

### Mass spectrometry analysis

The LC-MS/MS analysis was performed at the SPARC BioCentre, The Hospital for Sick Children, Toronto, Canada. Briefly, the bioactive fractions were reduced with dithiothreitol (DTT), the free cysteine residues alkylated with iodoactetamide and digested overnight with modified bovine trypsin (Promega, Madison, USA). The tryptic peptides were desalted and loaded onto a 50 cm × 75 μm ID column containing RSLC 2 μm C18 packing material (EASY-Spray, Thermo-Fisher, Odense, Denmark) with an integrated emitter. The peptides were eluted into a Q-Exactive hybrid mass spectrometer (Thermo-Fisher, San Jose, CA) using an Easy-Spray nLC 1000 chromatography system (Thermo-Fisher, Odense Denmark) with a 90-minute gradient from 0% to 35% acetonitrile in 0.1% formic acid. The mass spectrometer was operated in a data dependent mode with 1 MS followed by 10 MS/MS spectra. The MS was acquired with a resolution of 70,000 FWHM, a target of 1 × 10^6^ ions and a maximum scan time of 120 ms. The MS/MS scans were acquired with a resolution of 17,500 FWHM, a target of 1 × 10^6^ ions and a maximum scan time of 120 ms using relative collision energy of 27%. A dynamic exclusion time of 15 seconds was used for the MS/MS scans. The raw data files were acquired with XCalibur 2.2 (Thermo-Fisher Scientific) and processed with the Sequest search engine (Thermo-Fisher Scientific) using the UniProt canine database August 12, 2014 version with 28,460 entries and with X!-Tandem (Beavis Informatics, Winnipeg, Canada). The processed data was imported into Scaffold 3.2 (Proteome Software, Portland, OR). Peptides were considered to be identified if the Scaffold score exceeded the 0.1% false discovery rate (FDR) as determined by searching against the reversed UniProt canine database.

### Development and characterization of a pre-clinical rodent injury model of DDD

We used 12-week old female Wistar rats (n = 21, Charles River Laboratories Inc., MA, USA) to develop a pre-clinical rodent tail disc injury model of DDD as follows. Experimental protocols were approved by the Animal Care Committee and Research Ethics Approval Board of University Health Networks, Toronto, Ontario, Canada. All experimental procedures were performed in accordance with the guidelines approved by Animal Care Committee and Ethics Approval Board of University Health Networks, Toronto, Ontario, Canada. Anesthesia was achieved using isofluorane (5 L/min plus 1 L/min O_2_) and maintained at 3 L/min. Once deeply anaesthetized, the animal was affixed on a stereotactic procedure apparatus (Model 900, Kopf Instruments, CA, USA) with nose cone inhalation. For disc injury (4 caudal discs per animal), we used a 26-gauge (G) needle (Hamilton Company, USA) mounted on a Hamilton syringe. The needle was advanced completely through the selected tail IVD to penetrate the full thickness inclusive of the annulus fibrosus on both sides of the disc. Confirmation of needle placement was made using fluoroscopy and maintained in position for 2 minutes, withdrawn halfway to the center of the NP and left there for 1 minute and then slowly withdrawn completely over a 1-minute period. The animals were then removed from the stereotactic apparatus and allowed to recover in a warmed cage. At the end of study period i.e. 72 hrs - 10 weeks, animals were humanely euthanized using CO_2_ asphyxiation and each vertebral lumbar/caudal motion segment was dissected aseptically. Of the four injured IVDs, atleast one IVD per animal was fixed in formalin as a representative for histological analysis and rest of the injured IVD - nucleus pulposi were harvested and lysed in RIPA buffer (50 mM Tris, pH = 7.4, 150 mM NaCl, 1% NP-40 and protease inhibitor cocktail) for western blotting.

### Evaluation of NCCM and soluble factors as therapeutic agents in pre-clinical rodent model of DDD

We used a pre-clinical rat-tail disc injury model of DDD for evaluating the therapeutic potential of NCCM and soluble factors identified. For the 1^st^ set of experiments involving NCCM, animals (n = 6/group) were randomized four weeks post-injury and an intra-discal injection (~8 μL) of either concentrated NCCM (2.2 μg/μl) or the control medium was given under general anesthesia using fluoroscopic image guidance in 4 injured discs per animal as described earlier. For the 2^nd^ set of experiments involving growth factors including CTGF and TGFβ1, animals (n = 6/group) were randomized four weeks post-injury and an intra-discal injection (~8 μL) of either phosphate buffered saline (PBS, 1X, pH = 7.2), CTGF (100 ng/mL), TGFβ1 (10 ng/mL) or a combination of CTGF (100 ng/mL) and TGFβ1 (10 ng/mL) was given under general anesthesia using fluoroscopic image guidance in 4 injured discs per animal as described above. Further, a group of six animals were left uninjured that served as age matched healthy controls in each set of experiments. At the end of experiments (i.e. six weeks later), at least one IVD per animal was fixed in formalin as a representative for histological analysis. The remainder of the NPs harvested from rat caudal IVDs were pooled together according to their respective groups (healthy/treated) for lysis in RIPA lysis buffer for western blotting. Each of these experiments was repeated independently to ensure reproducibility.

### Histology and immunohistochemistry (IHC)

For formalin fixation, at least one IVD per animal (healthy/injured following treatment with NCCM, PBS, CTGF or TGFβ1 alone or in combination) was harvested for paraffin embedding. The tissues were fixed in 10% formalin followed by decalcification and paraffin embedding. Hemaetoxylin and eosin (H&E) and Safranin-O staining was performed in duplicates on paraffin-embedded sections (5 μm) to assess general morphology and proteoglycan content^21^. Histological grading of IVD-NP (injury followed by treatment) was carried out on the basis of NP-cellularity in H&E sections as described earlier^48^. Following H&E and Safranin O staining, serial tissue sections were deparaffinized in xylene, hydrated in gradient alcohol followed by antigen retrieval in Tris-EDTA buffer (pH = 9.0) for IHC. The sections were incubated with hydrogen peroxide (0.3% v/v) in methanol for 15 minutes, followed by blocking with 10% serum. Thereafter, the slides were incubated with either rabbit/goat polyclonal or mouse monoclonal primary antibodies overnight (O/N) at 4 °C. Protein expression was detected using respective secondary antibodies (rabbit/goat/mouse) from Vectastain ABC kit and diaminobenzidine (DAB) as a chromogen. In negative controls, the primary antibody was replaced by isotype-matched IgG. Immunohistochemistry was done in at least 3 different IVD sections per group. The bright field sections were evaluated by light microscopic examination using a ScanScope XT, Aperio Whole Slide Scanner and Nikon bright field microscope.

### Ingenuity Pathway Analysis (IPA)

Ingenuity Pathway Analysis (IPA, Ingenuity Systems, www.ingenuity.com) was used to identify the canonical pathways, major protein hubs and the associated networks involved in the maintenance of a healthy disc NP^48^. Of the 303 proteins identified in the bio-active fractions of NCCM, only 94 ECM proteins were used for network analysis. The protein accession numbers were used to determine IPA abbreviated names. These notations were then used to navigate the databases available online. The eligible proteins served as nodes for generating the canonical and novel proteins interaction networks. All networks were scored [−log (p-value)] based on the number of network eligible molecules contained in these networks. The higher the score, the lower was the probability of finding the observed number of network eligible molecules by random chance. The canonical pathways were scored based on their p-values, calculated using the right-tailed Fisher Exact Test; a p-value ≤ 0.05 indicates a statistically significant, non-random association.

### Cell culture, Western blotting and quantitative real time PCR (qPCR)

Healthy rat lumbar/caudal spine IVDs, injured tail discs and bovine caudal IVD NPs were removed aseptically. Bovine caudal disc NPs were obtained from six, 3-year old steers. Human degenerative disc nucleus pulposus tissues were obtained from patients (n = 3) undergoing discectomy or fusion surgery at Toronto Western Hospital, University Health Network (UHN), Toronto. For human degenerative disc NP samples used in this study, informed consent was obtained from all the patients prior to surgery. All human tissue samples were collected in accordance to the guidelines approved by the Research Ethics Board, Toronto Western Hospital, UHN, Toronto. The nucleus pulposus (NP) was separated and enzymatically digested according to our established methods^20^,^21^. The next day, the cells were filtered with a 70 μm cell strainer and cultured within a hypoxic incubator (NuAire, MN, USA) in 3.5% O_2_, 5% CO_2_, in Advanced Dulbecco’s modified Eagle’s medium (ADMEM) supplemented with 8% fetal bovine serum (FBS), penicillin and streptomycin (100 U/mL) until passage (P2) as described earlier^20^,^21^. The NP cells obtained from rat IVDs were pooled together for treatment with respective agents as follows. The cells were either cultured in serum free DMEM (no treatment controls) or treated with IL-1β (10 ng/mL), TNFα (50 ng/mL), CTGF (10–100 ng/mL), WISP2 (10–100 ng/mL) or TGFβ1 (5–20 ng/mL) for various time points (24 hrs–72 hrs) under hypoxic conditions. The effect of pro-inflammatory cytokines (IL-1β and TNFα) and soluble factors (CTGF/WISP2/TGFβ1) on expression of ECM genes and proteins in NP cells were determined using qPCR and Western blotting respectively (See Supplementary Data for details). For Western blotting, protein estimation was performed using Bradford assay (BioRad, CA). Briefly, equal amounts of whole cell or tissue lysates prepared using RIPA lysis buffer were subjected to Western blotting as described earlier^49^. Total lysates (30 μg) were resolved on 10% sodium dodecyl sulphate-polyacrylamide gels (SDS-PAGE) under reducing conditions and then proteins were electro-transferred onto polyvinyledendifluoride (PVDF) membranes (BioRad, CA). After blocking with 5% non-fat powdered milk in Tris-buffered saline (TBS, 0.1 M, pH = 7.4), blots were incubated with rabbit/goat polyclonal or mouse monoclonal primary antibodies at 4 °C overnight (See Supplementary Data for antibody details). Membranes were washed three times with Tween (0.1%)-Tris-buffer saline (TTBS) and then incubated for 2 hrs at room temperature (RT) with the respective HRP-conjugated anti-IgG secondary antibodies (BioRad, CA), diluted as per the manufacturers suggestions in 2% non-fat milk in TBS (pH = 7.2, 1X). Blots were washed three times with TTBS for 15 minutes and protein bands were detected by the enhanced chemiluminescence method (BioRad, CA) on Kodak Hyperfilm^50^. β-actin was used as a loading control for each experiment. All Western blots were run under the same experimental conditions and repeated at least 3 times to ensure reproducibility. All Western blots were quantified using densitometry values calculated using Image J software (available online). For calculation of fold changes representing expression levels of protein under each treatment conditions, densitometry values of each band for the protein of interest was normalized with β-actin used as a loading control in each experiment^50^.

### Cell viability, cell proliferation and apoptosis assays

Cell viability was determined using 3-(4,5-dimethylthiazol-2-yl)-2,5-diphenyltetrazolium bromide (MTT, Sigma-Aldrich, USA) as described earlier^50^. We used a BrdU - ELISA (colorimetric) assay (Cat# ab126556, Abcam) to determine the effect of treatment with CTGF (100 ng/mL) or TGFβ1 (10 ng/mL) on human and rat NP cells (healthy/injured) following the manufacturer’s instructions. Apoptosis in NP cells (rat and human) was determined using Caspase-3/7 and Caspase-9 specific Lumi-Glo assays (Promega, Madison). See Supplementary Data for details.

### Statistical analysis

All data for cell viability, proliferation, apoptosis (casase-3/7, caspase-9) assays and qPCR are expressed as means ± SD. Significant differences in test and no treatment controls (NTC) were determined using the paired Student’s t-test. Statistical analysis was performed using the Graphpad Prism. Data analysis for qPCR was carried out using calculation of ΔΔCt values, fold changes and p-values using Student’s t-test. P-value < 0.05 was defined as statistically significant for all tests. Ingenuity Pathway Analysis (IPA) software calculated p-values for canonical pathways based on the Fisher Exact Test; a p-value < 0.05 indicates a statistically significant, non-random association.

## Additional Information

**How to cite this article**: Matta, A. *et al*. Molecular Therapy for Degenerative Disc Disease: Clues from Secretome Analysis of the Notochordal Cell-Rich Nucleus Pulposus. *Sci. Rep.* **7**, 45623; doi: 10.1038/srep45623 (2017).

**Publisher's note:** Springer Nature remains neutral with regard to jurisdictional claims in published maps and institutional affiliations.

## Supplementary Material

Supplementary Data

## Figures and Tables

**Table 1:** Representative list of proteins identified in NCCM and their functions.

S. No.	Accession Number	Protein Name-FASTA	Function/Cell Signaling	References
1	F1PW10_CANFA (+1)	Transforming Growth Factor Beta-1	Involved in development of notochord, Stimulates ECM synthesis	24,26
2	J9NTX8_CANFA	Connective Tissue Growth Factor	Involved in development of notochord, Stimulates ECM synthesis, Regulates TGFβ signaling	27, 28, 29, 30, 31
3	E2RJ75_CANFA	WNT1 Inducible Signaling Pathway Protein 2(CCN5)	Negatively regulates TGFβ signaling	32
4	F1PM26_CANFA	CD109	Negatively regulates TGFβ signaling	34, 35, 36
5	F6V790_CANFA	CILP	Regulates TGFβ signaling	37
6	PGS2_CANFA	Decorin	Negatively regulates TGFβ signaling	38
7	E2RJE0_CANFA [2]	Cartilage Oligomeric Matrix Protein1	Regulates TGFβ signaling	39
8	J9P9E4_CANFA	sclerostin (BMP antagonist)	Negatively regulates TGFβ signaling	40
9	E2RBE4_CANFA (+1)	Chordin	Negatively regulates TGFβ signaling	40
10	F1PY69_CANFA (+1)	Follistatin-Like Protein 1	Regulates TGFβ signaling	51
11	F6XL96_CANFA	Von Willebrand Factor A Domain-Containing Protein 1	Promotes matrix mineralization through TGFβ signaling	52
12	E2R612_CANFA	EGF-Containing Fibulin-Like Extracellular Matrix Protein	Regulates cell proliferation and apoptosis	53
13	E2RNR0_CANFA	Osteoglycin	Involved in muscle and bone linkage. Activates Samd3/4 independent of TGFβ.	54
14	E2R8H9_CANFA (+1)	pleiotrophin, Neurite Growth-Promoting Factor 11	Involved in Angiogenesis and neurite growth	55
15	F1P7Y6_CANFA	nidogen-2	Involved in ECM assembly, cell invasion, involved in Angiogenesis	56
16	F1PQV4_CANFA	Olfactomedin-Like 3	Binds to BMP4 enhances the canonical SMAD1/5/8 signaling pathway, involved in Angiogenesis	57
17	F1PN55_CANFA	Frizzled-Related Protein 1	Regulates Wnt signalling	41
18	E2R2G1_CANFA	Angiopoietin-Like 7	Involved in Angiogenesis	45
19	E2R0R3_CANFA	Semaphorin	Inhibits angiogenesis and nerve ingrowth in IVD	46,47
20	F1Q3G2_CANFA	Slit Homolog 3 (Drosophila)	Involved in Angiogenesis	58
21	E2RMA3_CANFA	Secreted Protein, Acidic, Cysteine-Rich (Osteonectin)	Involved in ECM assembly, invasion and proliferation	59
22	E2R161_CANFA (+1)	Secreted Phosphoprotein 1 (Osteopontin)	Involved in ECM composition, invasion and migration	59
