# Supplementary material for: Molecular Therapy for Degenerative Disc Disease: Clues from Secretome Analysis of the Notochordal Cell-Rich Nucleus Pulposus
Source: Sci Rep. 2017 Mar 30;7:45623. doi: 10.1038/srep45623 (PMC5372366; doi:10.1038/srep45623)
Supplement: Supplementary Data [file srep45623-s1.pdf]

Title: Molecular Therapy for Degenerative Disc Disease: Clues from Secretome Analysis of the Notochordal Cell-Rich Nucleus Pulposus

Authors: Ajay Matta[1], M. Zia Karim[1], David E. Isenman[2] and W. Mark Erwin[1,3]*

Affiliations:

[1]Krembil Research Institute, Toronto Western Hospital, Toronto, Ontario, Canada.

[2]Department of Biochemistry, Faculty of Medicine, University of Toronto, Toronto, Ontario, Canada.

[3]Department of Surgery, University of Toronto, Toronto, Ontario, Canada.

Corresponding Author: *W. Mark Erwin DC, PhD, Krembil Research Institute, Toronto Western Hospital, Toronto, ON, Canada.

Email: mark.erwin@utoronto.ca; Tel: 416-603-5800 ext 3308

SUPPLEMENTARY DATA

MATERIALS AND METHODS

Antibodies and other reagents - Human recombinant interleukin-1β (IL-1β), tumor necrosis factor-alpha (TNFα), connective tissue growth factor (CTGF), Wnt–inducible soluble protein 2 (WISP2) and transforming growth factor beta 1 (TGFβ1) proteins were purchased from Peprotech Inc. (Quebec, Canada). We obtained rabbit polyclonal antibodies for Collagen 2 (ab34712), MMP-13 (ab39012), Cox2 (ab15191), Oct4 (ab18976), Nanog (ab106465), CTGF (ab6995), rabbit monoclonal for MMP-3 (ab52915) and mouse monoclonal antibodies for Galectin 3 (ab2785) and β-actin (ab6276) were purchased from Abcam Inc. (CA, USA). Goat polyclonal Brachyury antibody (sc-17743) and rabbit polyclonal antibodies for Aggrecan (sc-25674), TIMP-1 (sc-5538), ADAMTS-4 (sc-25582), TNFα (sc-8301), TGFβ1 (sc-146) and mouse monoclonal WISP2 (sc-514070) were obtained from Santa Cruz Biotechnology Inc. (CA, USA).

RNA isolation and Real Time PCR- Total RNA was isolated from human degenerative disc NP cells (H1 / H2 / H3) treated with IL-1β (10 ng/mL), TNFα (50 ng/mL), CTGF (100ng/mL), TGFβ1 (10 ng/mL) or a combination of CTGF (100 ng/mL) and TGFβ1 (10 ng/mL) using RNAeasy extraction kit (Cat#74134, Qiagen) and quantified using a Nanodrop spectrophotometer. Total RNA (~ 400ng) was reverse-transcribed using RT^2 First Strand Kit (Cat#330401, Qiagen) following the manufacturer's instructions for preparing cDNA. Effect of treatment on ECM genes including collagen 2, HAPLN1, versican, thrombospondin 1 (Thbs1), MMP-13 and Cox2 was evaluated using real time PCR performed on ABI 7900HT 384-well Fast block machine.

Cell viability and cell proliferation assays - Nucleus pulposus cells (rat, bovine and human) in passage (P2), were plated in 96-well flat bottom plates in order to evaluate the effect of treatment with growth factors in cell viability and proliferation assays. Rat NP cells (passage, P2) were treated with CTGF, WISP-2 and TGFβ1 in a dose (1 ng/mL – 100 ng/mL) and time dependent manner (24 hrs – 96 hrs) to determine the optimum dose and time for the assessment of these growth factors on viability. Cell viability was determined using 3-(4,5-dimethylthiazol-2-yl)-2,5-diphenyltetrazolium bromide (MTT, Sigma-Aldrich, USA) as described earlier[49]. We used a BrdU - ELISA (colorimetric) assay (Cat# ab126556, Abcam) for determining the effect of treatment with CTGF (100 ng/mL) or TGFβ1 (10 ng/mL) on human and rat NP cells (healthy / injured) following the manufacturer's instructions. Briefly, NP cells were treated with CTGF (100 ng/mL) or TGFβ1 (10 ng/mL) alone or in combination for 48 hrs following addition of BrdU reagent O/N in each well. Incorporated BrdU in DNA of proliferating cells was determined using anti-BrdU antibody and quantified by ELISA as per the manufacturer's instructions.

Apoptosis assays - Inflammation induced apoptosis in NP cells (rat and human) was determined using Caspase 3/7 and Caspase 9 specific Lumi-Glo assays (Promega, Madison). Briefly, NP cells were plated and treated with either IL-1β (10 ng/mL) alone or in combination with TNFα (50 ng/mL) only or in presence of CTGF (100 ng/mL) and TGFβ1 (10 ng/mL) for 48 hrs, followed by addition of specific reagents for Caspase 3/7 and Caspase 9 as per the manufacturer's instructions. Cells were incubated for another 4 hrs at 37^{0}C, and plates were read in a multi-well luminescence plate reader.

RESULTS

Histological analysis - Histology of the degenerative disc NPs obtained from human and bovine was compared to healthy NPs obtained from NCD canine and Wistar rat IVDs. Both human and bovine degenerative IVDs showed fibrocartilaginous matrix with strong Safranin-O staining in comparison to healthy, highly cellular NC - rich NP (>90%) with faint Safranin-O staining in NCD – canines and Wistar rat (Supplementary Fig. 2A). Immunohistochemical analysis revealed no detectable expression of Brachyury (NC–specific marker) or Oct4 (stem cell marker) in human and bovine degenerative disc NPs (Supplementary Fig.1A). In contrast, strong nuclear expression of brachyury and Oct4 was observed in healthy, NCD canine and Wistar rat discs demonstrating the NC- and stem cell-rich NP (Supplementary Fig. 2A). However, negative control tissue sections showed no immunostaining demonstrating the specificity of the antibodies (Supplementary Fig. 2B).

FIGURE LEGENDS

Supplementary Fig. 1. (A) Histological changes in rat disc NP in a time dependent manner (healthy – 10 weeks p.i.) with hematoxylin and eosin (H&E) staining showing loss of NCs and Safranin-O (Saf-O) staining showing development of fibrocartilaginous matrix (Scale bar 50μ). Panel (B) represents histograms showing fold changes in protein expression of IL1β, TNFα, Cox2, MMP-3, MMP-13, TIMP1 and ADAMTS-4. Panel (C) represents histograms showing fold changes in protein expression of Brachyury, Galectin3, Oct4 and Nanog. Each bar in the histogram represents mean ± S.D. of 3 independent experiments. *,**$p<0.05$; *p-values are w.r.t No treatment controls; **p-values are w.r.t 10 weeks p.i.

Supplementary Fig. 2. Panel (A) Safranin O staining and immunohistochemical analysis of brachyury and Oct4 proteins in paraffin embedded sections of nucleus pulposus obtained from human degenerative disc, bovine degenerative disc, non-chondrodystrophic canine and healthy rat discs (Scale bar 10μ). Arrows represent CLC, chondrocyte like cells and NC, notochordal cells. Panel (B) shows representative negative controls showing no immunostaining in tissue sections (rat healthy or mongrel IVD-NP), wherein specific rabbit or goat polyclonal antibodies were replaced by isotype controls (Scale bar 50μ). Panel (C) represents histograms showing fold changes in protein expression of Collagen2, Oct4 and Nanog in rat IVD-NP injected with PBS, control media or NCCM. Each bar in the histogram represents mean ± S.D. of 3 independent experiments. *,**$p<0.05$; *p-values are w.r.t No treatment controls; **p-values are w.r.t 10 weeks p.i.

Supplementary Fig. 3. Evaluation of bioactivity in protein containing fractions (PF) collected after size exclusion chromatography. Histograms showing (A) cell viability, (B) caspase-3/7

activity in bovine NP cells treated with NCCM, the cytotoxic drug, etoposide (300 μM, used as a positive control) in presence of NCCM or control medium (serum, phenol red and protein free Hybridoma Media). Each bar represents mean ± S.D. of 2 independent experiments done in triplicates (n=6). (C) Histograms showing caspase-3/7 activity in bovine NP cells treated with etoposide in control medium (serum free, phenol red free Hybridoma Media) or in presence of protein containing fractions (PFs). Each bar represents mean ± S.D. of 2 independent experiments done in triplicates (n=6). For these experiments, protein containing fractions (PFs) were mixed (1:1) with control medium (serum free, phenol red free Hybridoma Media) and compared to no treatment controls (i.e. elution buffer + control medium (1:1).

Supplementary Fig. 4. Ingenuity Pathway Analysis showing (A) Canonical signaling pathways identified. (B) Radial model showing CTGF as important hub proteins regulating / interacting with several other growth factors and ECM proteins. (C) Protein network involved in cartilage development and degradation; (D) and (E) Cell signaling involved in axonal growth. Proteins highlighted were identified in the secretome analysis.

Supplementary Fig. 5. Dose (5 ng/mL – 100 ng/mL) and time dependent (24 hrs - 96 hrs) effects of (A) CTGF, (B) WISP2 and (C) TGFβ1 treatment alone or (D) combination of CTGF with WISP2, and TGFβ1 on cell viability determined using MTT assays in NP cells obtained from healthy rat IVDs. Each bar represents mean ± S.D. of 3 independent experiments done in quadruplicates (n=12).

Supplementary Fig. 6. Effect of CTGF, WISP2 and TGFβ1 treatment alone or in combination on cell viability as determined using MTT assays in NP cells obtained from (A) rat (healthy / injured) disc, $*p \leq 0.02$, (B) bovine degenerative disc, $*p < 0.01$ and (C) human (H1, H2)

degenerative NP cells in 48 hrs (*p<0.005,**p=0.02). Each bar represents mean ± S.D. of 3 independent experiments done in triplicates (n=9).

Supplementary Fig. 7. Panel (A) shows a representative Western blot panel verifying increased collagen 2 expression in human degenerative disc NP cells(#H2) on treatment with CTGF and TGFβ1. Panel (B) represents histograms showing fold changes in protein expression of Collagen2 in human NP cells treated with CTGF, TGFβ1 alone or in combination as compared to no treatment controls (NTC).

Supplementary Fig. 8. Panel (A) represents histograms showing fold changes in protein expression of MMP-3, MMP-13 and Cox2 on treatment with IL1β alone or in presence of CTGF, WISP2 or TGFβ1. Panel (B) represents histograms showing fold changes in protein expression of MMP-3, MMP-13 and Cox2 on treatment with IL1β and TNFα alone or in presence of CTGF, WISP2 or TGFβ1. Each bar in the histogram represents mean ± S.D. of 5 independent experiments. *,**p<0.05; *p-values are w.r.t No treatment controls (NTC); **p-values are w.r.t IL1β or a combination of IL1β and TNFα.

Supplementary Fig. 9. Panel represents histograms showing fold change in protein expression of MMP-13, Cox2, Brachyury and Oct4 in rat IVD-NP injected with PBS, CTGF, TGFβ1 alone or in combination. **p<0.05; p-values are w.r.t PBS controls. Each bar in the histogram represents mean ± S.D. of 3 independent experiments.

Supplementary Fig. 1.

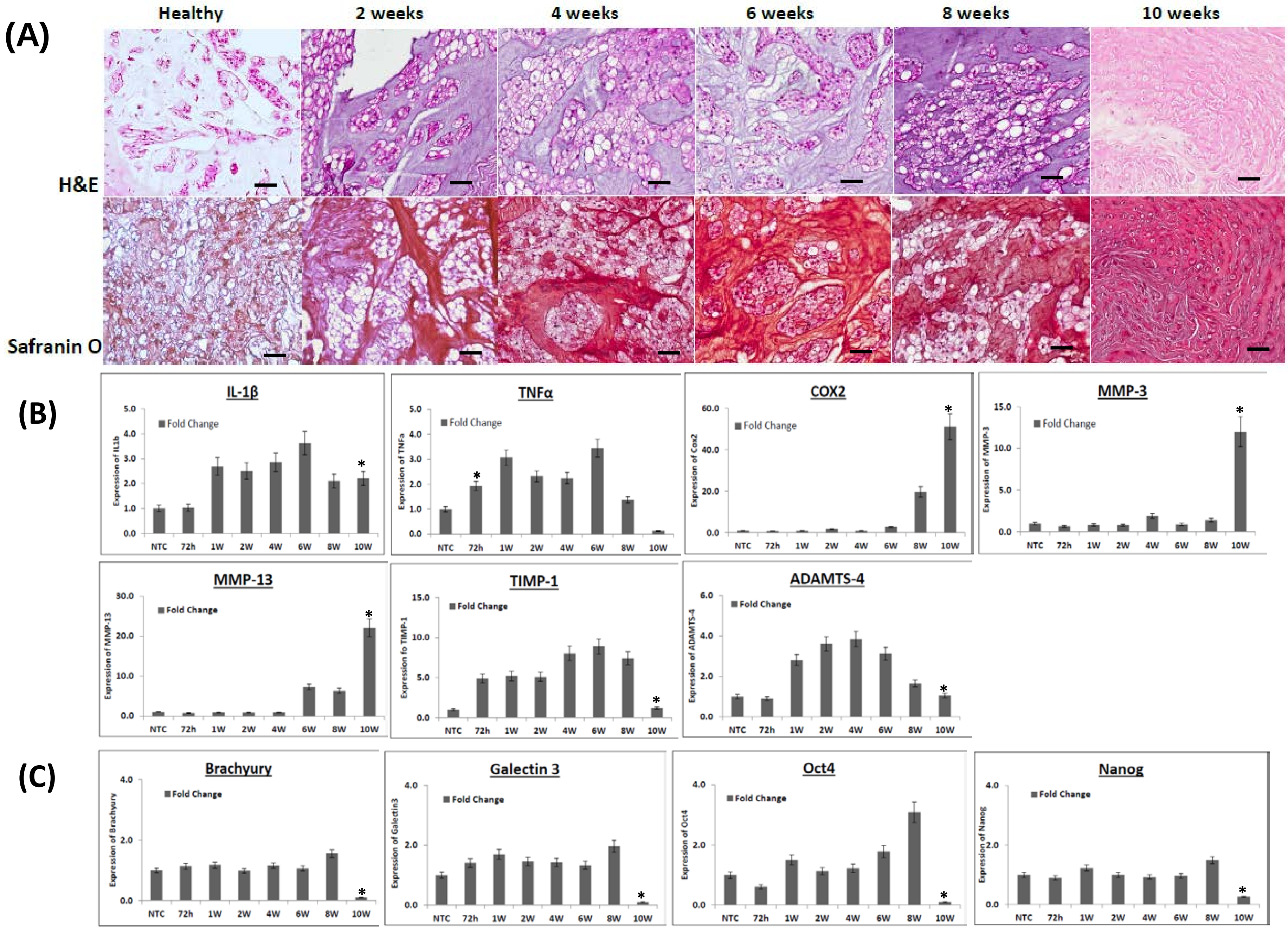

Supplementary Fig. 2.

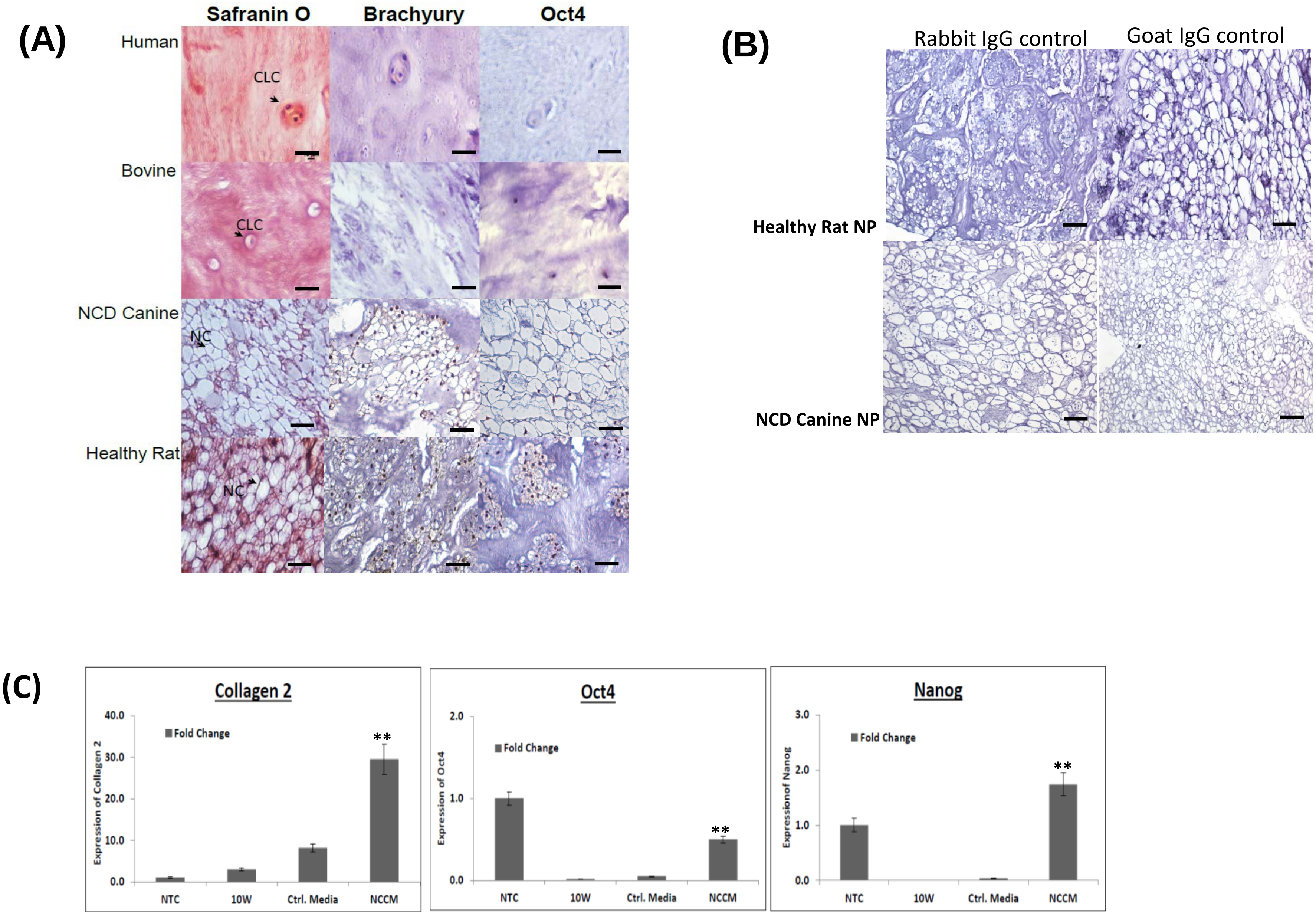

Supplementary Fig. 3.

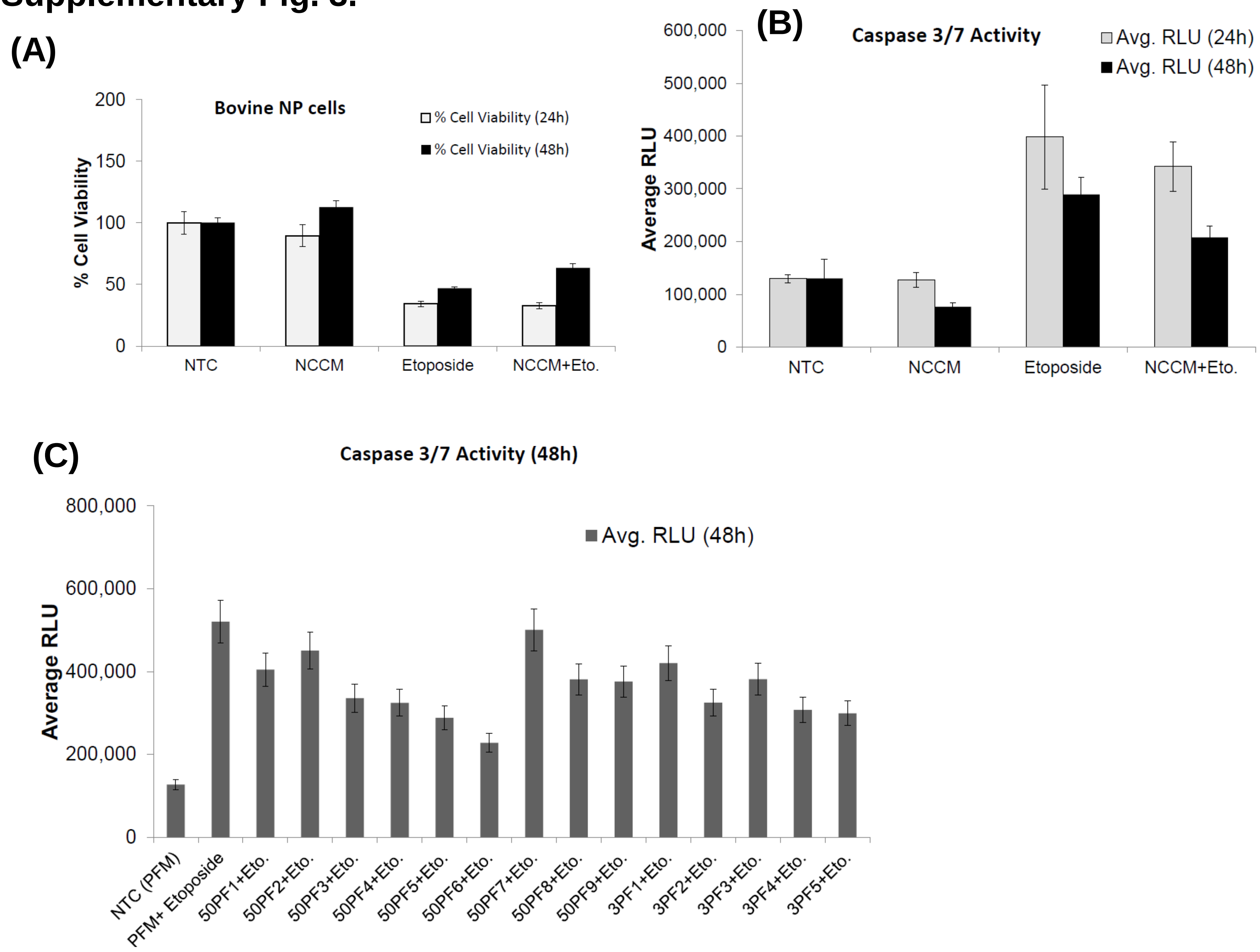

Supplementary Fig. 4.

(A)

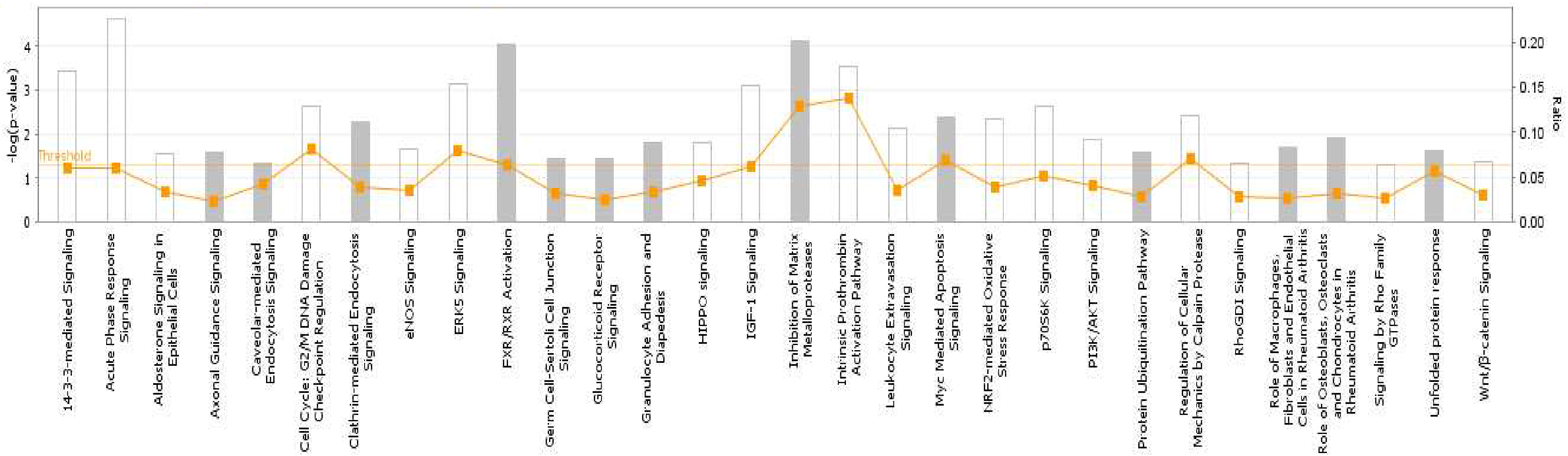

Supplementary Fig. 4.

(B)

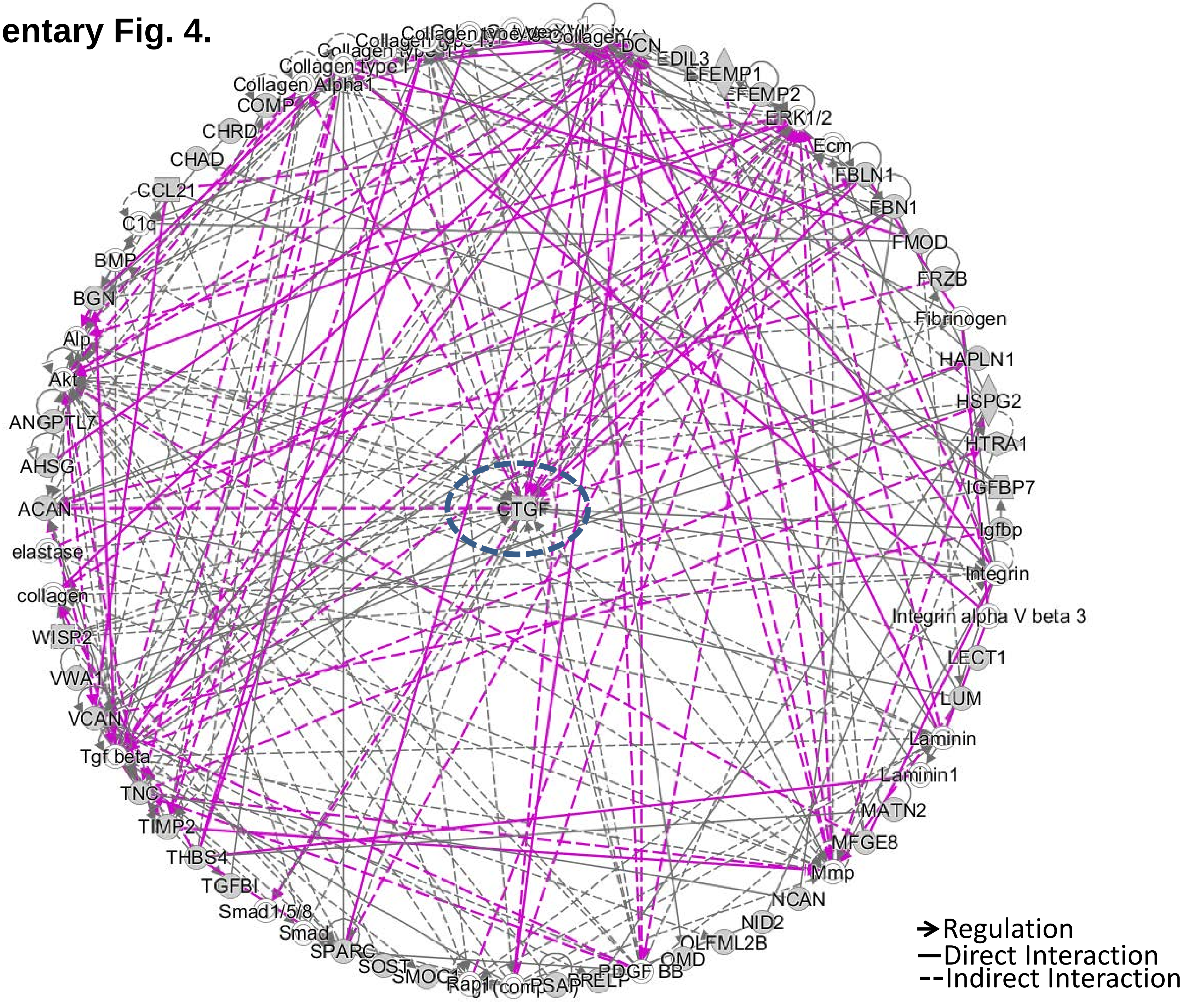

Supplementary Fig. 4.

(C)

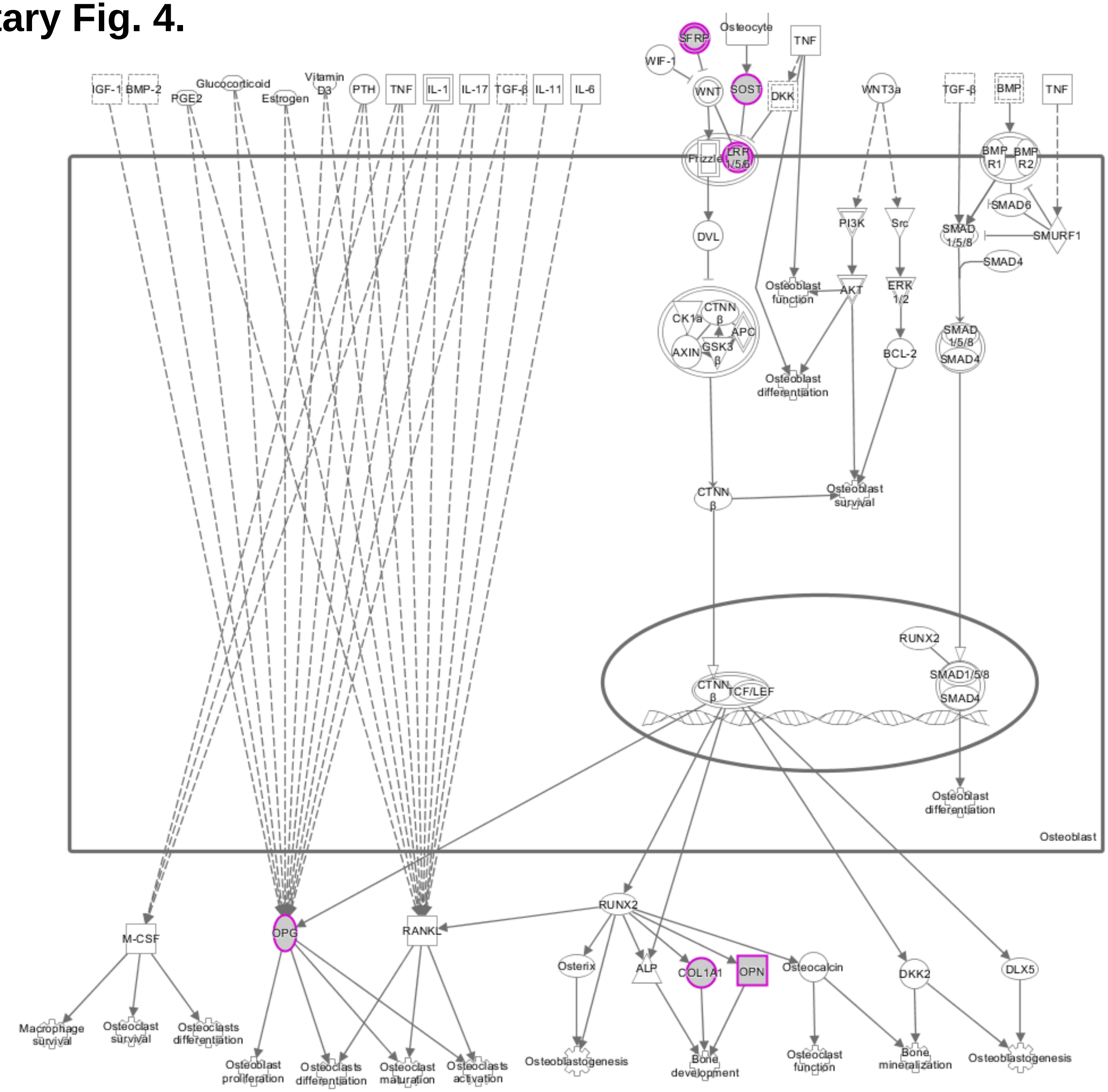

Supplementary Fig. 4.

(D)

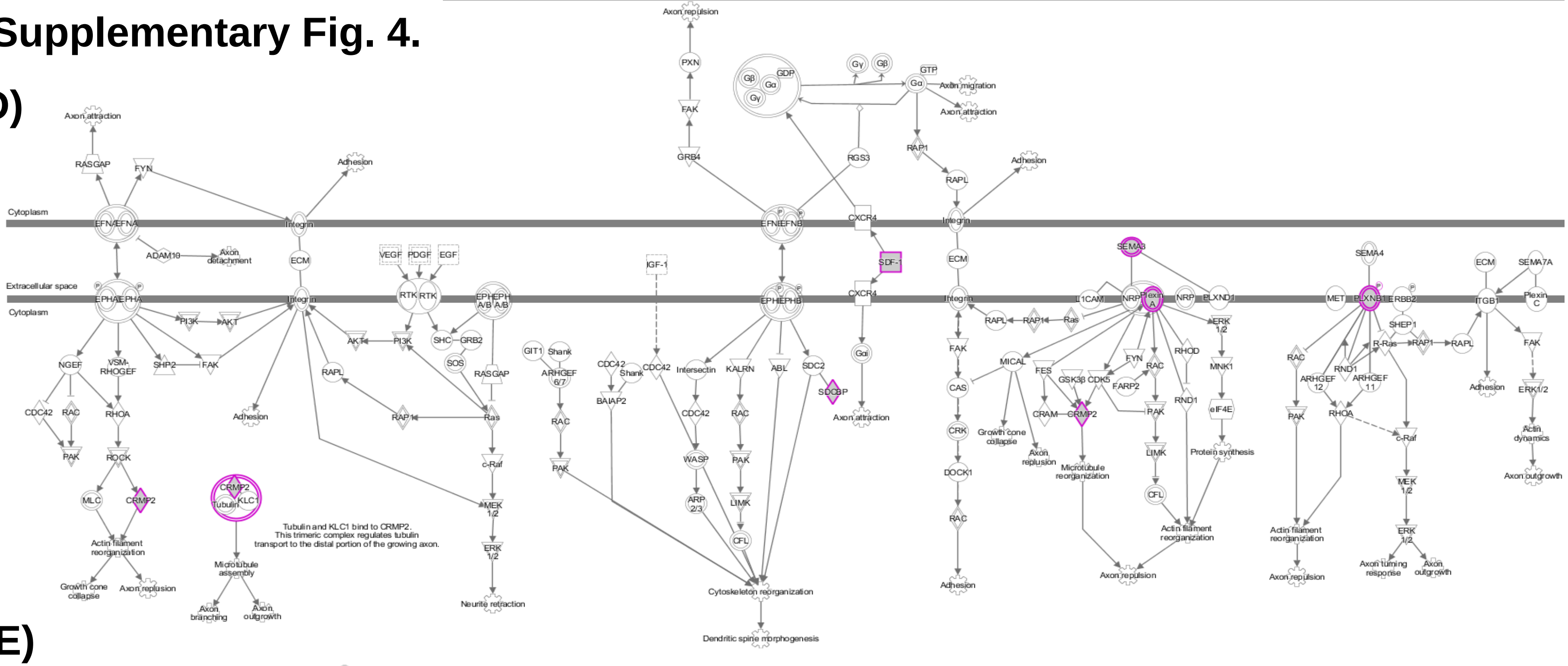

(E)

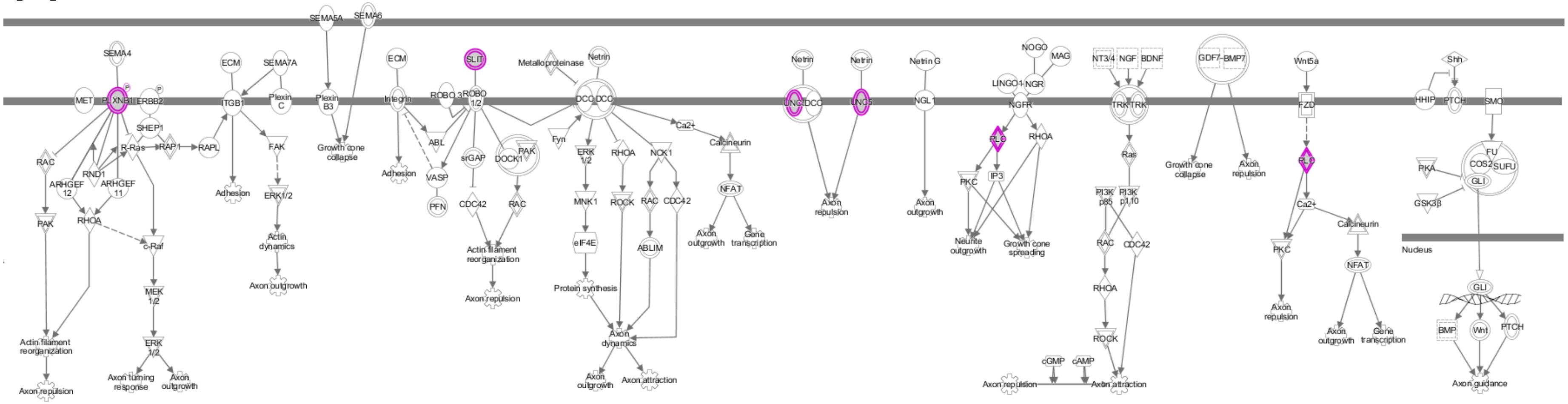

Supplementary Fig. 5.

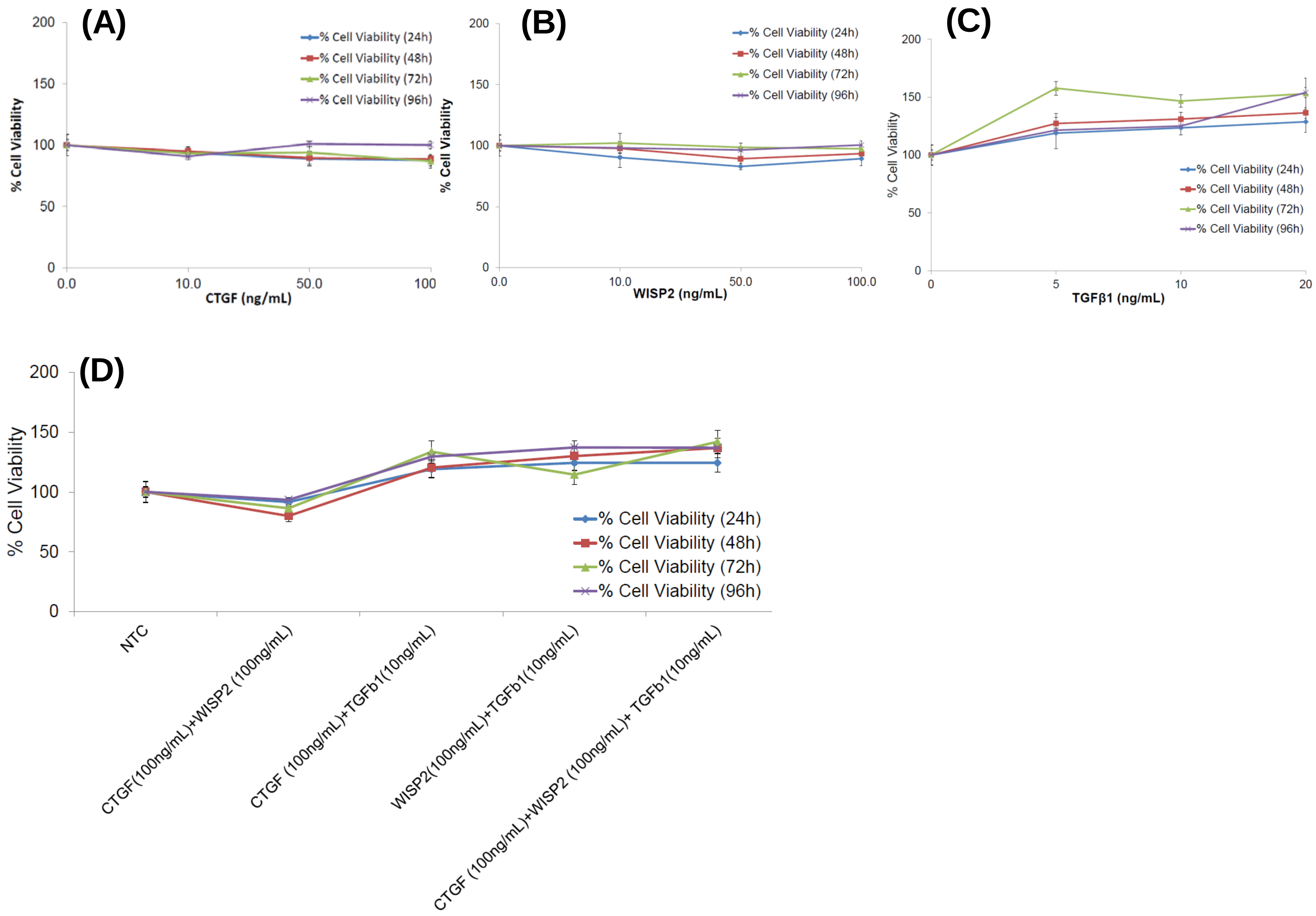

Supplementary Fig. 6.

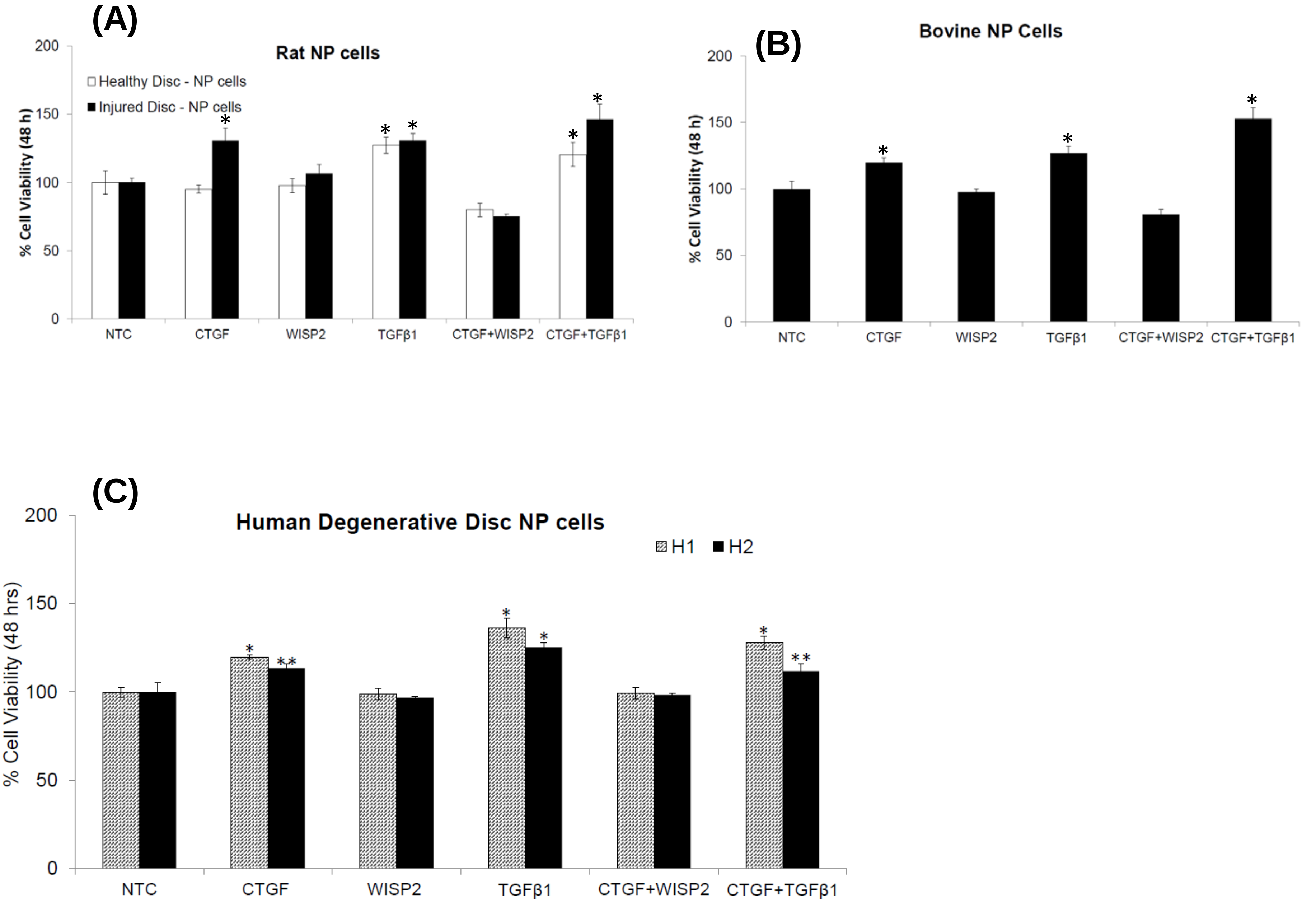

Supplementary Fig. 7.

(A)

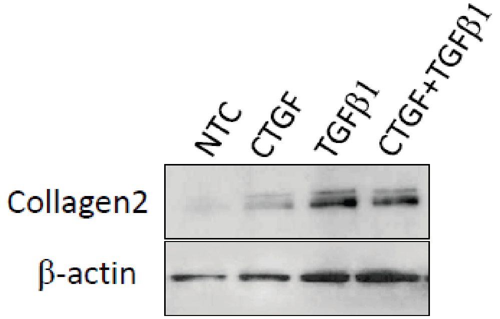

(B)

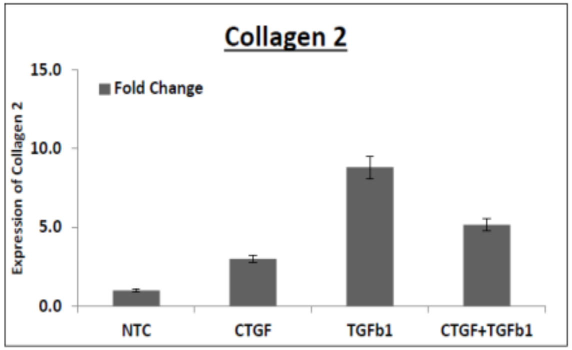

Supplementary Fig. 8.

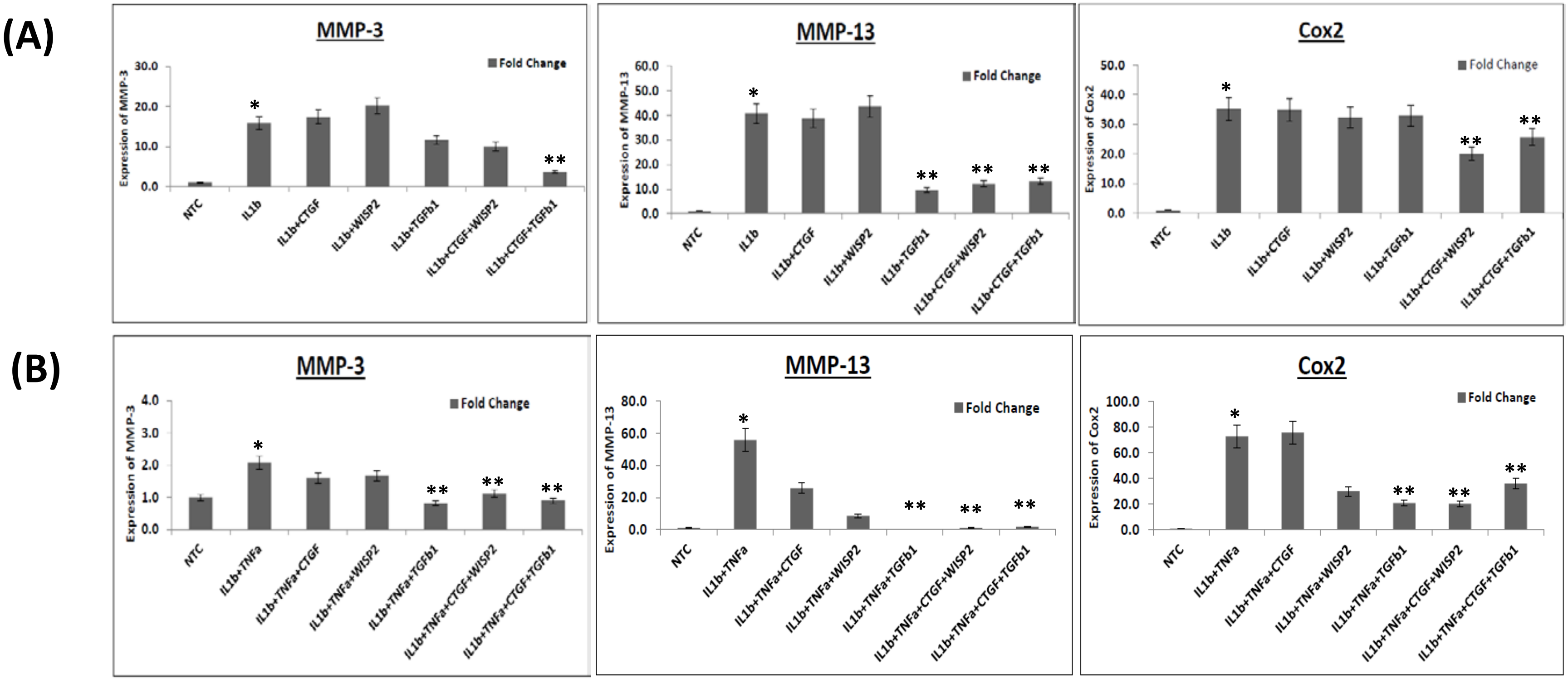

Supplementary Fig. 9.

(A)

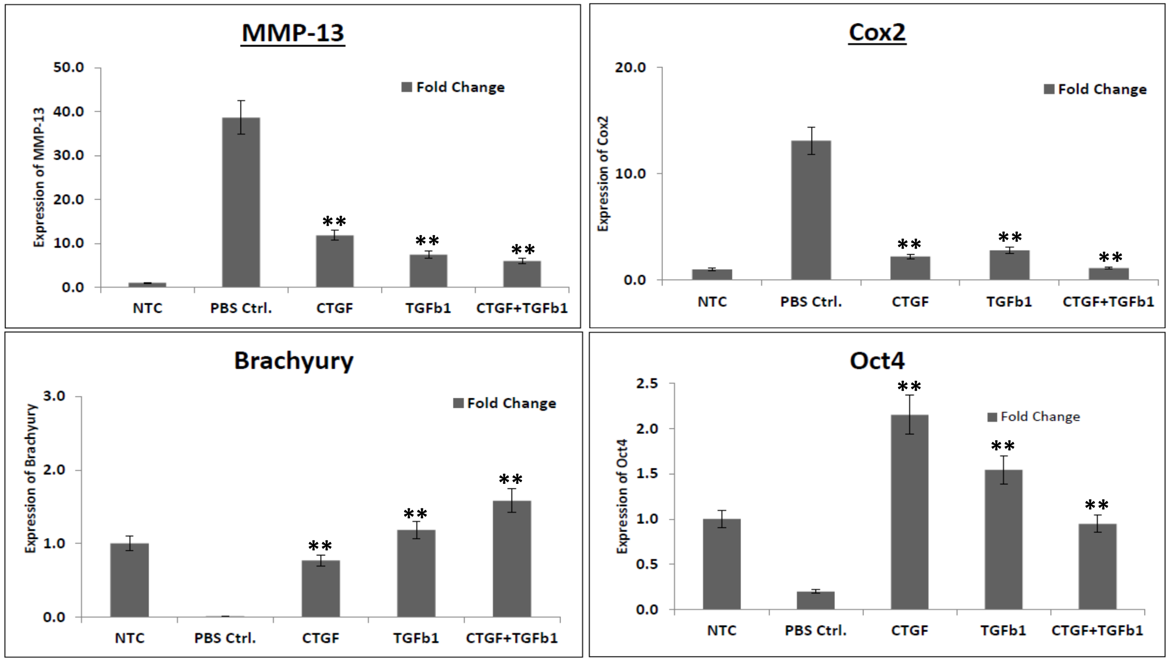

Supplementary Table S1. List of proteins identified in NCCM

S. No.	Identified Proteins	Accession Number	Protein Name-FASTA
1	Uncharacterized protein (Fragment) OS=Canis familiaris GN=TGFBI PE=4 SV=2	F1PW10_CANFA (+1)	Transforming Growth Factor Beta-1
2	Uncharacterized protein OS=Canis familiaris GN=CTGF PE=4 SV=1	J9NTX8_CANFA	Connective Tissue Growth Factor
3	Uncharacterized protein OS=Canis familiaris GN=WISP2 PE=4 SV=2	E2RJ75_CANFA	WNT1 Inducible Signaling Pathway Protein 2(CCN5)
4	Uncharacterized protein OS=Canis familiaris GN=SOST PE=4 SV=1	J9P9E4_CANFA	sclerostin (BMP antagonist)
5	Chordin OS=Canis familiaris GN=CHRD PE=3 SV=2	E2RBE4_CANFA (+1)	Chordin
6	Uncharacterized protein (Fragment) OS=Canis familiaris GN=FSTL1 PE=4 SV=2	F1PY69_CANFA (+1)	Follistatin-Like Protein 1
7	Uncharacterized protein OS=Canis familiaris GN=CD109 PE=4 SV=2	F1PM26_CANFA	CD109
8	Uncharacterized protein (Fragment) OS=Canis familiaris GN=FRZB PE=4 SV=2	F1PN55_CANFA	Frizzled-Related Protein 1
9	Uncharacterized protein OS=Canis familiaris GN=VWA1 PE=4 SV=1	F6XL96_CANFA	Von Willebrand Factor A Domain-Containing Protein 1
10	Uncharacterized protein OS=Canis familiaris GN=IGFBP7 PE=4 SV=1	E2RNL2_CANFA	Insulin-Like Growth Factor Binding Protein 7
11	Uncharacterized protein OS=Canis familiaris GN=IGFL3 PE=4 SV=1	E2QTE1_CANFA	Insulin Growth Factor-Like Family Member 3
12	Cation-independent mannose-6-phosphate/insulin-like growth factor 2 receptor protein OS=Canis familiaris GN=CI-MPR/IGF2R PE=2 SV=1	B1H0W0_CANFA (+1)	Cation-independent mannose-6-phosphate/insulin-like growth factor 2 receptor protein
13	Uncharacterized protein (Fragment) OS=Canis familiaris GN=EGFR PE=3 SV=2	F1PF03_CANFA	EGFR
14	Uncharacterized protein OS=Canis familiaris GN=EFEMP1 PE=4 SV=1	E2R612_CANFA	EGF-Containing Fibulin-Like Extracellular Matrix Protein
15	Uncharacterized protein OS=Canis familiaris GN=EDIL3 PE=4 SV=2	F1P8D5_CANFA	EGF-Like Repeat And Discoidin I-Like Domain-Containing Protein 32
16	Uncharacterized protein OS=Canis familiaris GN=EFEMP2 PE=4 SV=1	E2RNX7_CANFA	EGF-Containing Fibulin-Like Extracellular Matrix Protein 2
17	Uncharacterized protein (Fragment) OS=Canis familiaris GN=OLFML2B PE=4 SV=2	F1PAD2_CANFA	Olfactomedin-Like Protein 2B
18	Uncharacterized protein OS=Canis familiaris GN=OLFML3 PE=4 SV=2	F1PQV4_CANFA	Olfactomedin-Like 3
19	Uncharacterized protein OS=Canis familiaris GN=OGN PE=4 SV=2	E2RNR0_CANFA	Osteoglycin
20	Uncharacterized protein OS=Canis familiaris GN=SPARC PE=4 SV=1	E2RMA3_CANFA	Secreted Protein, Acidic, Cysteine-Rich (Osteonectin)
21	Uncharacterized protein OS=Canis familiaris GN=SPP1 PE=4 SV=1	E2R161_CANFA (+1)	Secreted Phosphoprotein 1 (Osteopontin)
22	Uncharacterized protein (Fragment) OS=Canis familiaris GN=PRPH PE=3 SV=2	F1PGH6_CANFA	Peripherin
23	Uncharacterized protein OS=Canis familiaris GN=GFAP PE=3 SV=1	E2RS09_CANFA	Glial Fibrillary Acidic Protein1
24	Uncharacterized protein OS=Canis familiaris GN=ANGPTL7 PE=4 SV=1	E2R2G1_CANFA	Angiopoietin-Like 7
25	Uncharacterized protein OS=Canis familiaris GN=SLIT3 PE=3 SV=2	F1Q3G2_CANFA	Slit Homolog 3 (Drosophila)
26	Uncharacterized protein (Fragment) OS=Canis familiaris GN=ROR2 PE=4 SV=2	F1PSD7_CANFA	Receptor Tyrosine Kinase-Like Orphan Receptor 2
27	Uncharacterized protein OS=Canis familiaris GN=PTN PE=4 SV=1	E2R8H9_CANFA (+1)	pleiotrophin, Neurite Growth-Promoting Factor 11
28	Uncharacterized protein OS=Canis familiaris GN=LRP1 PE=4 SV=2	E2R4Q1_CANFA (+1)	Low Density Lipoprotein Receptor-Related Protein 1
29	Uncharacterized protein OS=Canis familiaris GN=VLDLR PE=4 SV=2	F1P787_CANFA	Very Low Density Lipoprotein Receptor
30	Uncharacterized protein (Fragment) OS=Canis familiaris GN=LRP1B PE=4 SV=2	F1PNZ9_CANFA (+1)	Low Density Lipoprotein Receptor-Related Protein 1B
31	Uncharacterized protein (Fragment) OS=Canis familiaris GN=LRP2 PE=4 SV=2	F1PIB4_CANFA	Low Density Lipoprotein Receptor-Related Protein 2
32	Uncharacterized protein OS=Canis familiaris GN=THY1 PE=4 SV=1	J9P7X2_CANFA	CD90
33	Metalloproteinase inhibitor 1 OS=Canis familiaris GN=TIMP1 PE=4 SV=2	F1PQS2_CANFA (+1)	TIMP1
34	Metalloproteinase inhibitor 2 (Fragment) OS=Canis familiaris GN=TIMP2 PE=4 SV=2	F1Q4F9_CANFA	TIMP2
35	Timp3 OS=Canis familiaris GN=timp3 PE=2 SV=1	F5CBP3_CANFA (+1)	Timp3
36	Uncharacterized protein (Fragment) OS=Canis familiaris GN=COL15A1 PE=4 SV=1	F1PS66_CANFA (+1)	COL15A1

Supplementary Table S1 contd.

S. No.	Identified Proteins	Accession Number	Protein Name-FASTA
**37**	Cluster of Uncharacterized protein OS=Canis familiaris GN=COL6A3 PE=4 SV=2 (F1PR31_CANFA)	F1PR31_CANFA [3]	COL6A3
**38**	Uncharacterized protein OS=Canis familiaris GN=COL6A1 PE=4 SV=2	F1PFM6_CANFA	COL6A1
**39**	Uncharacterized protein OS=Canis familiaris GN=COL2A1 PE=4 SV=1	F1PS24_CANFA (+1)	COL2A1
**40**	Cluster of Uncharacterized protein OS=Canis familiaris GN=COL11A2 PE=4 SV=1 (F1PQD5_CANFA)	F1PQD5_CANFA [2]	COL11A2
**41**	Uncharacterized protein OS=Canis familiaris GN=COL12A1 PE=4 SV=1	F6XWS5_CANFA (+1)	COL12A1
**42**	Uncharacterized protein OS=Canis familiaris GN=COL3A1 PE=4 SV=2	F1PG69_CANFA (+1)	COL3A1
**43**	PE=1 SV=1	CO1A1_CANFA (+1)	Collagen alpha-1
**44**	Uncharacterized protein (Fragment) OS=Canis familiaris GN=COL11A1 PE=4 SV=2	F1PD36_CANFA (+1)	COL11A1
**45**	Uncharacterized protein OS=Canis familiaris GN=COL5A2 PE=4 SV=2	F1PG08_CANFA	COL5A2
**46**	Uncharacterized protein OS=Canis familiaris GN=PCOLCE2 PE=4 SV=1	E2R7L9_CANFA (+2)	Procollagen C-Proteinase Enhancer 1
**47**	Aggrecan core protein OS=Canis familiaris GN=ACAN PE=4 SV=1	F1PWT9_CANFA	Aggrecan core protein
**48**	Fibronectin OS=Canis familiaris GN=FN1 PE=4 SV=1	J9P8M2_CANFA	Fibronectin
**49**	Uncharacterized protein (Fragment) OS=Canis familiaris GN=HSPG2 PE=4 SV=1	J9NRJ0_CANFA	Heparan Sulfate Proteoglycan Core Protein, Perlecan
**50**	Uncharacterized protein OS=Canis familiaris GN=LUM PE=4 SV=1	E2R416_CANFA	Lumican Proteoglycan
**51**	Uncharacterized protein OS=Canis familiaris GN=PRG4 PE=4 SV=2	F1PXD6_CANFA	Proteoglycan 4
**52**	Uncharacterized protein OS=Canis familiaris GN=CHAD PE=4 SV=1	E2QRS3_CANFA	Chondroadherin Proteoglycan, Cartilage Leucine-Rich Protein
**53**	Uncharacterized protein OS=Canis familiaris GN=VCAN PE=4 SV=2	F1PGL1_CANFA	Versican
**54**	Decorin OS=Canis familiaris GN=DCN PE=2 SV=2	PGS2_CANFA	Decorin
**55**	Uncharacterized protein OS=Canis familiaris GN=HAPLN1 PE=4 SV=1	J9PAT9_CANFA	HAPLN1
**56**	Biglycan OS=Canis familiaris GN=BGN PE=4 SV=1	G1K2D8_CANFA (+1)	Biglycan
**57**	Uncharacterized protein OS=Canis familiaris GN=FMOD PE=4 SV=2	F1PLN5_CANFA	Fibromodulin
**58**	Uncharacterized protein (Fragment) OS=Canis familiaris GN=TNC PE=4 SV=1	F1PF31_CANFA (+1)	TNC
**59**	Uncharacterized protein OS=Canis familiaris GN=GSN PE=4 SV=1	F6Y3P9_CANFA	Gelsolin
**60**	Uncharacterized protein OS=Canis familiaris GN=CSPG4 PE=4 SV=1	J9P6X3_CANFA	Chondroitin Sulfate Proteoglycan 4
**61**	Uncharacterized protein OS=Canis familiaris GN=THBS4 PE=4 SV=2	F1PSS2_CANFA	Thrombospondin 4
**62**	Uncharacterized protein OS=Canis familiaris GN=PRELP PE=4 SV=1	E2RL80_CANFA	Proline-Arginine-Rich End Leucine-Rich Repeat Protein2,Prolargin Proteoglycan1
**63**	Uncharacterized protein OS=Canis familiaris GN=OMD PE=4 SV=2	E2RNR9_CANFA (+1)	osteomodulin, Keratan Sulfate Proteoglycan Osteomodulin2
**64**	Cluster of Uncharacterized protein OS=Canis familiaris GN=NEBL PE=4 SV=2 (F1PQ88_CANFA)	F1PQ88_CANFA [2]	Nebulette
**65**	Uncharacterized protein OS=Canis familiaris GN=PSAP PE=4 SV=1	E2RLF1_CANFA	prosaposin
**66**	Fibulin-1 OS=Canis familiaris GN=FBLN1 PE=3 SV=2	F1PLV6_CANFA	Fibulin-1
**67**	Uncharacterized protein OS=Canis familiaris GN=ABI3BP PE=4 SV=2	F1PKX2_CANFA	ABI Gene Family, Member 3 (NESH) Binding Protein2
**68**	Uncharacterized protein OS=Canis familiaris GN=NID2 PE=4 SV=2	F1P7Y6_CANFA	nidogen-2
**69**	Uncharacterized protein OS=Canis familiaris GN=SEMA3C PE=4 SV=2	E2R0R3_CANFA	Semaphorin
**70**	Uncharacterized protein OS=Canis familiaris GN=SEMA3E PE=4 SV=2	F1Q4C1_CANFA	semaphorin-3E
**71**	Cluster of Uncharacterized protein OS=Canis familiaris GN=COMP PE=4 SV=2 (E2RJE0_CANFA)	E2RJE0_CANFA [2]	Cartilage Oligomeric Matrix Protein1
**72**	Uncharacterized protein OS=Canis familiaris GN=CILP2 PE=4 SV=2	E2RCP2_CANFA	Cartilage Intermediate Layer Protein-Like Protein 2
**73**	Uncharacterized protein OS=Canis familiaris GN=CILP PE=4 SV=1	F6V790_CANFA	CILP

Supplementary Table 1 contd.

S. No.	Identified Proteins	Accession Number	Protein Name-FASTA
74	Galectin OS=Canis familiaris GN=LGALS3 PE=2 SV=1	E5Q8W5_CANFA (+2)	Galectin
75	Uncharacterized protein (Fragment) OS=Canis familiaris GN=CLSTN1 PE=4 SV=1	F1PE64_CANFA	Calsyntenin 1
76	Uncharacterized protein OS=Canis familiaris GN=LOXL3 PE=4 SV=1	E2QYZ4_CANFA	LOXL3
77	Uncharacterized protein OS=Canis familiaris GN=SMOC1 PE=4 SV=2	F1P8D9_CANFA	SPARC-Related Modular Calcium-Binding Protein 1
78	Uncharacterized protein OS=Canis familiaris GN=SDCBP PE=4 SV=2	F1PKR0_CANFA	Syndecan Binding Protein (Syntenin)
79	Dystroglycan OS=Canis familiaris GN=DAG1 PE=3 SV=1	DAG1_CANFA	Dystroglycan
80	Clusterin OS=Canis familiaris GN=CLU PE=2 SV=1	CLUS_CANFA	Clusterin
81	Desmin OS=Canis familiaris GN=DES PE=3 SV=3	DESM_CANFA	Desmin
82	Uncharacterized protein (Fragment) OS=Canis familiaris GN=GPC1 PE=3 SV=1	F1PPA1_CANFA	Glypican Proteoglycan 1
83	Cluster of Uncharacterized protein (Fragment) OS=Canis familiaris GN=VCL PE=4 SV=2 (F1PKR3_CANFA)	F1PKR3_CANFA [2]	vinculin
84	Uncharacterized protein OS=Canis familiaris GN=FBN1 PE=4 SV=2	F1PIA3_CANFA	Fibrillin 1
85	Uncharacterized protein OS=Canis familiaris GN=MATN2 PE=4 SV=2	F1PGH3_CANFA (+1)	Matrilin 2
86	Uncharacterized protein OS=Canis familiaris GN=VIT PE=4 SV=2	E2QY57_CANFA (+1)	VIT
87	Tumor necrosis factor receptor superfamily member 11B OS=Canis familiaris GN=TNFRSF11B PE=3 SV=1	F6V4E0_CANFA	Tumor necrosis factor receptor superfamily member 11B
88	Cluster of Uncharacterized protein OS=Canis familiaris GN=LYPD8 PE=4 SV=1 (F6XCE8_CANFA)	F6XCE8_CANFA [2]	Ly6/PLAUR Domain-Containing Protein 8
89	Uncharacterized protein OS=Canis familiaris GN=CLEC3A PE=4 SV=1	E2RQC0_CANFA	C-Type (Calcium Dependent, Carbohydrate-Recognition Domain) Lectin,Superfamily Member 1 (Cartilage-Derived)1
90	Uncharacterized protein OS=Canis familiaris GN=LGALS3BP PE=4 SV=1	E2RKQ6_CANFA	Lectin Galactoside-Binding Soluble 3-Binding Protein2
91	Uncharacterized protein OS=Canis familiaris GN=CCL16 PE=4 SV=1	J9NV49_CANFA	IL-10-Inducible Chemokine
92	Uncharacterized protein (Fragment) OS=Canis familiaris GN=MFGE8 PE=4 SV=2	F1PFZ5_CANFA	Milk Fat Globule-EGF Factor 8
93	Uncharacterized protein OS=Canis familiaris GN=CCDC80 PE=4 SV=2	E2RGR7_CANFA	Coiled-Coil Domain-Containing Protein 80
94	Rab GDP dissociation inhibitor beta (Fragment) OS=Canis familiaris GN=GDI2 PE=4 SV=1	F1P8L7_CANFA	Rab GDP dissociation inhibitor beta
95	Uncharacterized protein (Fragment) OS=Canis familiaris GN=PTGFRN PE=4 SV=2	F1PR26_CANFA	Prostaglandin F2 Receptor Inhibitor
96	Uncharacterized protein (Fragment) OS=Canis familiaris GN=HTRA1 PE=4 SV=2	F1PU95_CANFA	HtrA Serine Peptidase 1
97	Uncharacterized protein OS=Canis familiaris GN=KAL1 PE=4 SV=2	F1Q1G0_CANFA	Kallmann Syndrome 1 Sequence
98	Uncharacterized protein OS=Canis familiaris GN=TSPAN8 PE=4 SV=1	E2QSC8_CANFA	tetraspanin-8
99	Uncharacterized protein OS=Canis familiaris GN=PARK7 PE=4 SV=1	E2QS13_CANFA	Parkinson Protein 7
100	Sulfhydryl oxidase (Fragment) OS=Canis familiaris GN=QSOX1 PE=3 SV=2	F1PLT8_CANFA	Sulfhydryl oxidase
101	CC chemokine ligand 21 OS=Canis familiaris GN=CCL21 PE=2 SV=1	Q68AM9_CANFA	CCL21
102	Uncharacterized protein OS=Canis familiaris GN=WDR1 PE=2 SV=2	F1PR93_CANFA	WD Repeat-Containing Protein 1
103	Uncharacterized protein OS=Canis familiaris GN=CDH2 PE=3 SV=2	F1PEC4_CANFA	CDH2
104	Cadherin-1 OS=Canis familiaris GN=CDH1 PE=1 SV=2	CADH1_CANFA (+1)	Cadherin-1
105	Uncharacterized protein (Fragment) OS=Canis familiaris GN=NCAM1 PE=4 SV=2	F1PMU2_CANFA (+3)	Neural Cell Adhesion Molecule 1
106	Uncharacterized protein OS=Canis familiaris GN=BCAM PE=4 SV=2	F1PWJ7_CANFA (+1)	Basal Cell Adhesion Molecule
107	Uncharacterized protein (Fragment) OS=Canis familiaris GN=NCAN PE=4 SV=2	E2RBU8_CANFA	neurocan (Chondroitin Sulfate Proteoglycan 3)
108	Trappin-2 OS=Canis familiaris GN=PI3 PE=4 SV=1	D3DMB4_CANFA	Trappin
109	Galectin OS=Canis familiaris GN=LGALS1 PE=2 SV=1	E2RJL1_CANFA (+1)	Galectin
110	Uncharacterized protein OS=Canis familiaris GN=XIRP2 PE=4 SV=2	F1PCJ4_CANFA-DECOY (·	Xin Actin-Binding Repeat-Containing Protein 2
111	Uncharacterized protein OS=Canis familiaris GN=CD55 PE=4 SV=1	J9NTG7_CANFA	Decay Accelerating Factor For Complement (CD55,

Supplementary Table S1 contd.

S. No.	Identified Proteins	Accession Number	Protein Name-FASTA
**112**	Uncharacterized protein OS=Canis familiaris GN=GM2A PE=4 SV=2	F1P8U3_CANFA	GM2 Ganglioside Activator
**113**	Uncharacterized protein (Fragment) OS=Canis familiaris GN=CXCL12 PE=4 SV=2	F1PNB2_CANFA	CXCL12
**114**	Uncharacterized protein OS=Canis familiaris GN=LECT2 PE=4 SV=1	E2RCY6_CANFA	Leukocyte Cell-Derived Chemotaxin-2
**115**	Uncharacterized protein OS=Canis familiaris GN=EHD3 PE=4 SV=2	F1PXX1_CANFA	EH-Domain Containing 3
**116**	Uncharacterized protein OS=Canis familiaris GN=FOLR2 PE=4 SV=1	E2QXF0_CANFA	Folate Receptor 2 (Fetal)
**117**	Uncharacterized protein OS=Canis familiaris GN=PDCD6 PE=4 SV=2	F1Q1H3_CANFA	Programmed Cell Death 6
**118**	Uncharacterized protein OS=Canis familiaris GN=SSC5D PE=4 SV=2	E2R0D2_CANFA	Scavenger Receptor Cysteine Rich Domain Containing (5 Domains)
**119**	Uncharacterized protein OS=Canis familiaris GN=EML2 PE=4 SV=2	E2RII4_CANFA (+1)	Echinoderm Microtubule Associated Protein Like 2
**120**	Uncharacterized protein OS=Canis familiaris GN=FAM3C PE=4 SV=2	F1PGP3_CANFA	Family With Sequence Similarity 3, Member C
**121**	Uncharacterized protein OS=Canis familiaris GN=CD63 PE=4 SV=2	F1PIT4_CANFA	Tetraspanin-30
**122**	Uncharacterized protein OS=Canis familiaris GN=S100A6 PE=4 SV=1	E2R5P5_CANFA	S100A6
**123**	Uncharacterized protein OS=Canis familiaris GN=S100A13 PE=4 SV=1	E2R9P2_CANFA	S100A13
**124**	Uncharacterized protein OS=Canis familiaris GN=LAMP1 PE=4 SV=2	F1Q260_CANFA	Lysosome-Associated Membrane Protein 1
**125**	Uncharacterized protein OS=Canis familiaris GN=GAS1 PE=4 SV=1	E2R2S1_CANFA	Growth Arrest-Specific 1
**126**	Cathepsin L1 OS=Canis familiaris GN=CTSL PE=2 SV=1	CATL1_CANFA	Cathepsin L1
**127**	Uncharacterized protein OS=Canis familiaris GN=SCRN2 PE=4 SV=1	E2QTH4_CANFA	Secernin 2
**128**	Uncharacterized protein OS=Canis familiaris GN=UNC5C PE=4 SV=2	F1PUV2_CANFA (+1)	Netrin Receptor UNC5C
**129**	Uncharacterized protein (Fragment) OS=Canis familiaris GN=AEBP1 PE=4 SV=2	H9GW59_CANFA	AE Binding Protein 1
**130**	Epididymal secretory protein E1 (Fragment) OS=Canis familiaris GN=NPC2 PE=4 SV=2	F1PAR9_CANFA (+1)	Epididymal secretory protein E1
**131**	Uncharacterized protein OS=Canis familiaris GN=LAMP2 PE=4 SV=2	E2RNJ1_CANFA (+1)	Lysosome-Associated Membrane Protein 2
**132**	Cluster of Uncharacterized protein OS=Canis familiaris GN=PLXNA3 PE=4 SV=2 (F1PWQ0_CANFA)	F1PWQ0_CANFA [3]	plexin-A3
**133**	Uncharacterized protein OS=Canis familiaris GN=PLXNB2 PE=4 SV=2	F1P9S5_CANFA	Plexin B2
**134**	Uncharacterized protein OS=Canis familiaris GN=PLXDC2 PE=4 SV=2	F1PNK7_CANFA	Plexin Domain Containing 2
**135**	Uncharacterized protein OS=Canis familiaris GN=EHD2 PE=4 SV=2	F1PNP7_CANFA	EH-Domain Containing 2
**136**	Uncharacterized protein OS=Canis familiaris GN=AHSG PE=4 SV=2	E2QUV3_CANFA	alpha-2-HS-glycoprotein
**137**	Uncharacterized protein OS=Canis familiaris GN=CAPG PE=4 SV=2	E2R413_CANFA	Gelsolin-Like Capping Protein
**138**	Mucin OS=Canis familiaris PE=2 SV=1	Q28226_CANFA	Mucin
**139**	Uncharacterized protein OS=Canis familiaris GN=SLC29A1 PE=3 SV=2	E2RLS3_CANFA	Solute Carrier Family 29 (Nucleoside Transporters), Member 1
**140**	Uncharacterized protein OS=Canis familiaris GN=CA2 PE=4 SV=2	F1PDY8_CANFA	CA2
**141**	Uncharacterized protein OS=Canis familiaris GN=CA3 PE=4 SV=2	F1PDZ7_CANFA	Carbonic Anhydrase 3
**142**	Uncharacterized protein OS=Canis familiaris GN=PLOD1 PE=4 SV=2	F1P9Z8_CANFA (+1)	Procollagen-Lysine, 2-Oxoglutarate 5-Dioxygenase 1
**143**	Uncharacterized protein (Fragment) OS=Canis familiaris GN=PAM PE=4 SV=1	F1Q4G4_CANFA	Peptidylglycine Alpha-Amidating Monooxygenase
**144**	Uncharacterized protein OS=Canis familiaris GN=ECHDC1 PE=3 SV=2	F1PW22_CANFA	Enoyl-CoA Hydratase Domain-Containing Protein 1
**145**	Uncharacterized protein (Fragment) OS=Canis familiaris GN=PCSK5 PE=3 SV=2	F1P9I5_CANFA (+2)	Proprotein Convertase Subtilisin/Kexin Type 5
**146**	Uncharacterized protein (Fragment) OS=Canis familiaris GN=CHST6 PE=4 SV=2	E2RSN9_CANFA	Carbohydrate (N-Acetylglucosamine 6-O) Sulfotransferase 6
**147**	Uncharacterized protein OS=Canis familiaris GN=APLP2 PE=4 SV=1	E2RPM5_CANFA (+1)	Amyloid Beta (A4) Precursor-Like Protein 2

Supplementary Table S1 contd.

S. No.	Identified Proteins	Accession Number	Protein Name-FASTA
**148**	Uncharacterized protein OS=Canis familiaris GN=GBE1 PE=4 SV=2	F1PX32_CANFA	Glucan (1,4-Alpha-), Branching Enzyme 1
**149**	Beta-glucuronidase OS=Canis familiaris GN=GUSB PE=1 SV=1	BGLR_CANFA (+1)	Beta-glucuronidase
**150**	Xylosyltransferase 1 (Fragment) OS=Canis familiaris GN=XYLT1 PE=4 SV=1	F1PB62_CANFA (+1)	Xylosyltransferase 1
**151**	Uncharacterized protein OS=Canis familiaris GN=GPLD1 PE=4 SV=2	E2RK02_CANFA	Glycosylphosphatidylinositol Specific Phospholipase D11
**152**	Uncharacterized protein OS=Canis familiaris GN=SIAE PE=4 SV=2	F1PXN3_CANFA	Sialic Acid Acetylesterase1
**153**	Carbohydrate sulfotransferase-like protein OS=Canis familiaris GN=CHST3 PE=4 SV=2	E2RLE9_CANFA	Carbohydrate sulfotransferase-like protein
**154**	N-acetylgalactosamine-6-sulfatase (Fragment) OS=Canis familiaris GN=GALNS PE=4 SV=2	F1PHF0_CANFA (+1)	N-acetylgalactosamine-6-sulfatase
**155**	Uncharacterized protein OS=Canis familiaris GN=MLXIPL PE=4 SV=1	E2RAQ3_CANFA	MLX Interacting Protein-Like
**156**	Uncharacterized protein OS=Canis familiaris GN=LYZ PE=3 SV=1	G1K265_CANFA (+1)	Lyzozyme
**157**	Serum albumin OS=Canis familiaris GN=ALB PE=1 SV=3	ALBU_CANFA	Serum albumin
**158**	Uncharacterized protein OS=Canis familiaris GN=SPTBN1 PE=4 SV=2	F1PXT8_CANFA (+1)	Spectrin, Beta, Non-Erythrocytic 1
**159**	Histone H4 OS=Canis familiaris GN=HIST1H4L PE=3 SV=1	F2Z4N2_CANFA (+3)	Histone H4
**160**	Uncharacterized protein OS=Canis familiaris GN=HPX PE=4 SV=2	F1PZR4_CANFA	hemopexin
**161**	Uncharacterized protein OS=Canis familiaris GN=TF PE=4 SV=1	J9P430_CANFA	transferrin
**162**	Apolipoprotein E OS=Canis familiaris GN=APOE PE=3 SV=1	F1PJ74_CANFA	Apolipoprotein E
**163**	Uncharacterized protein OS=Canis familiaris GN=A2M PE=4 SV=1	F6UME0_CANFA	alpha-2-macroglobulin
**164**	Hemoglobin subunit alpha OS=Canis familiaris GN=HBA PE=1 SV=1	HBA_CANFA (+3)	Hemoglobin subunit alpha
**165**	Protein disulfide-isomerase OS=Canis familiaris GN=PDIA3 PE=3 SV=1	E2RD86_CANFA	Protein disulfide-isomerase
**166**	Uncharacterized protein OS=Canis familiaris GN=NPEPPS PE=4 SV=2	F1P7M0_CANFA	Aminopeptidase Puromycin Sensitive
**167**	Uncharacterized protein OS=Canis familiaris GN=PGAM4 PE=3 SV=2	E2RT65_CANFA	Phosphoglycerate Mutase Processed Protein
**168**	Cyclic nucleotide gated channel beta 1 OS=Canis familiaris GN=CNGB1 PE=2 SV=1	S4V0G9_CANFA (+1)	Cyclic nucleotide gated channel beta 1
**169**	Uncharacterized protein OS=Canis familiaris GN=UBB PE=4 SV=2	F1PDG4_CANFA (+3)	Ubiquitin B1
**170**	Cluster of Superoxide dismutase [Cu-Zn] (Fragment) OS=Canis familiaris GN=SOD1 PE=3 SV=2 (F1Q462_CANFA)	F1Q462_CANFA [2]	Superoxide dismutase [Cu-Zn]
**171**	Purine nucleoside phosphorylase OS=Canis familiaris GN=PNP PE=3 SV=2	F1PQM1_CANFA	Purine nucleoside phosphorylase
**172**	Uncharacterized protein (Fragment) OS=Canis familiaris GN=PGM1 PE=3 SV=2	F1PUL4_CANFA	Phosphoglucomutase 1
**173**	Triosephosphate isomerase OS=Canis familiaris GN=TPI1 PE=1 SV=3	TPIS_CANFA	Triosephosphate Isomerase
**174**	Aldose 1-epimerase OS=Canis familiaris GN=GALM PE=3 SV=1	E2QWA2_CANFA (+1)	Aldose 1-epimerase
**175**	Proteasome subunit alpha type OS=Canis familiaris GN=PSMA6 PE=3 SV=1	E2RMN2_CANFA	Proteasome subunit alpha
**176**	Fructose-bisphosphate aldolase OS=Canis familiaris GN=ALDOC PE=3 SV=1	F1PL63_CANFA (+1)	Fructose-bisphosphate aldolase
**177**	Uncharacterized protein OS=Canis familiaris GN=PGLS PE=4 SV=1	E2RLQ6_CANFA	6-phosphogluconolactonase1
**178**	Uncharacterized protein (Fragment) OS=Canis familiaris GN=DNAH10 PE=4 SV=2	E2RMJ8_CANFA	DNAH10
**179**	Uncharacterized protein OS=Canis familiaris GN=BLVRB PE=4 SV=1	E2QVU9_CANFA	Biliverdin Reductase B (Flavin Reductase (NADPH))
**180**	Uncharacterized protein OS=Canis familiaris GN=GPI PE=3 SV=1	E2R2C3_CANFA	Glucose Phosphate Isomerase
**181**	Fructose-bisphosphate aldolase (Fragment) OS=Canis familiaris GN=ALDOA PE=3 SV=2	F1PBT3_CANFA	Fructose-bisphosphate aldolase
**182**	Uncharacterized protein (Fragment) OS=Canis familiaris GN=HSP90AA1 PE=4 SV=2	F1PGY1_CANFA	HSP90AA1

Supplementary Table S1 contd.

S. No.	Identified Proteins	Accession Number	Protein Name-FASTA
183	Cluster of Uncharacterized protein OS=Canis familiaris GN=HSPA8 PE=3 SV=1 (E2R0T6_CANFA)	E2R0T6_CANFA [3]	HSPA8
184	Uncharacterized protein OS=Canis familiaris GN=HSPA8 PE=3 SV=1	E2R0T6_CANFA (+1)	HSPA8
185	Uncharacterized protein OS=Canis familiaris GN=HSPA2 PE=3 SV=1	E2QX84_CANFA	HSPA2
186	Annexin OS=Canis familiaris GN=ANXA5 PE=3 SV=2	E2RQ14_CANFA	ANXA5
187	Cluster of Uncharacterized protein OS=Canis familiaris GN=ACTN4 PE=4 SV=1 (E2R5T9_CANFA)	E2R5T9_CANFA [3]	ACTN4
188	Uncharacterized protein OS=Canis familiaris GN=ACTN4 PE=4 SV=1	E2R5T9_CANFA (+1)	ACTN4
189	Uncharacterized protein OS=Canis familiaris GN=ACTN1 PE=4 SV=2	E2QY08_CANFA	ACTN1
190	Annexin A2 OS=Canis familiaris GN=ANXA2 PE=1 SV=1	ANXA2_CANFA	ANXA2
191	Annexin OS=Canis familiaris GN=ANXA4 PE=3 SV=2	F1PXG4_CANFA	ANXA4
192	Annexin OS=Canis familiaris GN=ANXA8L1 PE=3 SV=2	E2R0S6_CANFA	Annexin
193	Glyceraldehyde-3-phosphate dehydrogenase (Fragment) OS=Canis familiaris GN=GAPDH PE=3 SV=2	F1PTZ9_CANFA	Glyceraldehyde-3-phosphate dehydrogenase
194	Uncharacterized protein OS=Canis familiaris GN=LAMB2 PE=4 SV=1	E2R4J1_CANFA	Laminin, Beta 2
195	Uncharacterized protein OS=Canis familiaris GN=LAMC1 PE=4 SV=2	F1PHK9_CANFA	Laminin B2 Chain
196	Uncharacterized protein OS=Canis familiaris GN=LAMA4 PE=4 SV=2	F1PR66_CANFA	Laminin, Alpha 4
197	L-lactate dehydrogenase OS=Canis familiaris GN=LDHA PE=3 SV=2	F1PVW0_CANFA	LDHA
198	Uncharacterized protein (Fragment) OS=Canis familiaris GN=MFI2 PE=4 SV=2	F1P7Y8_CANFA	melanotransferrin
199	Superoxide dismutase [Cu-Zn] OS=Canis familiaris GN=SOD3 PE=3 SV=2	F1PAS8_CANFA	Superoxide dismutase [Cu-Zn]
200	Uncharacterized protein OS=Canis familiaris GN=CP PE=4 SV=2	F1PAX2_CANFA	Ceruloplasmin (Ferroxidase)
201	Cluster of Uncharacterized protein OS=Canis familiaris GN=YWHAZ PE=3 SV=2 (F1PBL1_CANFA)	F1PBL1_CANFA [6]	YWHAZ
202	Amyloid beta A4 protein OS=Canis familiaris GN=APP PE=4 SV=2	F1P603_CANFA (+5)	Amyloid beta A4 protein
203	Uncharacterized protein OS=Canis familiaris GN=YWHAZ PE=3 SV=2	F1PBL1_CANFA	YWHAZ
204	Uncharacterized protein OS=Canis familiaris GN=YWHAB PE=3 SV=2	F1PKW7_CANFA	YWHAB
205	Uncharacterized protein (Fragment) OS=Canis familiaris GN=YWHAG PE=3 SV=1	J9NRH5_CANFA	YWHAG
206	Uncharacterized protein OS=Canis familiaris GN=YWHAH PE=3 SV=1	J9P6N4_CANFA	YWHAH
207	Uncharacterized protein OS=Canis familiaris GN=YWHAG PE=4 SV=1	E2QVP6_CANFA	YWHAG
208	Cluster of Actin, cytoplasmic 1 OS=Canis familiaris GN=ACTB PE=2 SV=3 (ACTB_CANFA)	ACTB_CANFA [2]	ACTB
209	Actin, cytoplasmic 1 OS=Canis familiaris GN=ACTB PE=2 SV=3	ACTB_CANFA	ACTB
210	Uncharacterized protein OS=Canis familiaris GN=ACTG1 PE=3 SV=2	F1PQL8_CANFA	ACTG1
211	Uncharacterized protein (Fragment) OS=Canis familiaris GN=TUBA1C PE=3 SV=2	E2RNQ2_CANFA (+1)	Tubulin, Alpha 1c
212	Uncharacterized protein OS=Canis familiaris GN=LTF PE=4 SV=1	F1PR54_CANFA	lactotransferrin
213	Phosphatidylethanolamine-binding protein 1 OS=Canis familiaris GN=PEBP1 PE=1 SV=1	PEBP1_CANFA	Phosphatidylethanolamine-binding protein 1
214	Alpha 1 antitrypsin OS=Canis familiaris PE=2 SV=1	A1ILJ0_CANFA (+1)	Alpha 1 antitrypsin
215	Uncharacterized protein OS=Canis familiaris GN=MRC2 PE=4 SV=2	F1PCR2_CANFA (+1)	Mannose Receptor, C Type 2
216	Malic enzyme OS=Canis familiaris GN=ME1 PE=3 SV=1	J9NZX7_CANFA	Malic enzyme
217	Uncharacterized protein OS=Canis familiaris GN=PRDX2 PE=4 SV=2	F1PCG4_CANFA	PRDX2
218	Cluster of Uncharacterized protein OS=Canis familiaris GN=GSTM4 PE=3 SV=2 (F1P6R7_CANFA)	F1P6R7_CANFA [3]	GSTM4

Supplementary Table S1 contd.

S. No.	Identified Proteins	Accession Number	Protein Name-FASTA
219	Uncharacterized protein OS=Canis familiaris GN=GSTM4 PE=3 SV=2	F1P6R7_CANFA (+1)	GSTM4
220	Uncharacterized protein OS=Canis familiaris GN=GSTM3 PE=3 SV=1	E2R5B9_CANFA	GSTM3
221	Uncharacterized protein OS=Canis familiaris GN=DPYSL2 PE=4 SV=2	F1P9U4_CANFA	Dihydropyrimidinase-Like 2
222	Uncharacterized protein OS=Canis familiaris GN=PRDX1 PE=4 SV=1	E2RHG2_CANFA	PRDX1
223	Uncharacterized protein OS=Canis familiaris GN=HSPA5 PE=3 SV=2	F1PIC7_CANFA	HSPA5
224	Annexin OS=Canis familiaris GN=ANXA1 PE=3 SV=1	F1P6B7_CANFA	Annexin
225	L-lactate dehydrogenase OS=Canis familiaris GN=LDHB PE=3 SV=1	E2R761_CANFA (+1)	L-lactate dehydrogenase
226	Phosphoglycerate kinase OS=Canis familiaris GN=PGK1 PE=3 SV=1	E2RRC9_CANFA	PGK1
227	Malate dehydrogenase (Fragment) OS=Canis familiaris GN=MDH1 PE=3 SV=2	E2QV08_CANFA (+1)	Malate dehydrogenase
228	Pyruvate kinase OS=Canis familiaris GN=PKM PE=3 SV=2	F1PHR2_CANFA	Pyruvate kinase
229	Uncharacterized protein OS=Canis familiaris GN=CALM1 PE=4 SV=1	E2REK6_CANFA	CALM1
230	Cluster of Uncharacterized protein OS=Canis familiaris GN=VIM PE=3 SV=1 (F1PLS4_CANFA)	F1PLS4_CANFA [17]	Vimentin
231	Cluster of Uncharacterized protein OS=Canis familiaris GN=EZR PE=4 SV=1 (E2RSI6_CANFA)	E2RSI6_CANFA	Ezrin
232	Uncharacterized protein OS=Canis familiaris GN=PLEC PE=4 SV=2	F1PHS5_CANFA	plectin
233	Uncharacterized protein (Fragment) OS=Canis familiaris GN=GALNT2 PE=3 SV=2	F1P835_CANFA (+1)	Polypeptide N-Acetylgalactosaminyltransferase 2
234	Cluster of Nucleoside diphosphate kinase (Fragment) OS=Canis familiaris GN=NME2 PE=3 SV=2 (E2RC20_CANFA)	E2RC20_CANFA [3]	Nucleoside diphosphate kinase
235	Uncharacterized protein (Fragment) OS=Canis familiaris GN=HSP70 PE=3 SV=1	G1K268_CANFA (+1)	HSP70
236	Annexin OS=Canis familiaris GN=ANXA11 PE=3 SV=1	E2QXN8_CANFA	Annexin
237	Ribonuclease A B1 OS=Canis familiaris GN=RNASE4 PE=3 SV=2	F1PPZ2_CANFA	Ribonuclease A B1
238	Uncharacterized protein OS=Canis familiaris GN=GSTP1 PE=3 SV=2	F1Q0J0_CANFA	GSTP1
239	Uncharacterized protein (Fragment) OS=Canis familiaris GN=UGP2 PE=4 SV=2	E2R5R9_CANFA (+1)	Uridyl Diphosphate Glucose Pyrophosphorylase 2
240	Uncharacterized protein (Fragment) OS=Canis familiaris GN=PRDX6 PE=4 SV=1	F1PC59_CANFA	PRDX6
241	Anionic trypsinogen (Fragment) OS=Canis familiaris PE=3 SV=1	A0FGS8_CANFA (+1)	Anionic trypsinogen
242	Transaldolase OS=Canis familiaris GN=LOC100855463 PE=3 SV=1	H9GW87_CANFA	Transaldolase
243	Elongation factor 1-alpha OS=Canis familiaris PE=3 SV=1	J9NU04_CANFA	Elongation factor 1-alpha
244	Uncharacterized protein OS=Canis familiaris GN=LECT1 PE=4 SV=2	F1PB82_CANFA	Leukocyte Cell Derived Chemotaxin 1
245	Uncharacterized protein OS=Canis familiaris GN=AKR1A1 PE=4 SV=2	F1PK43_CANFA	Aldo-Keto Reductase Family 1, Member A1 (Aldehyde Reductase)
246	Uncharacterized protein OS=Canis familiaris GN=SMPDL3A PE=4 SV=2	E2RRU2_CANFA (+1)	Sphingomyelin Phosphodiesterase, Acid-Like 3A
247	Uncharacterized protein OS=Canis familiaris GN=LMNA PE=3 SV=1	F1PBJ3_CANFA (+1)	Lamin A/C
248	Peptidyl-prolyl cis-trans isomerase OS=Canis familiaris GN=LOC100688474 PE=3 SV=1	F1PK62_CANFA (+1)	Peptidyl-prolyl cis-trans isomerase
249	Uncharacterized protein OS=Canis familiaris GN=ENO2 PE=3 SV=2	E2RAS8_CANFA	Enolase 2
250	Uncharacterized protein OS=Canis familiaris GN=TKT PE=4 SV=2	F1PE28_CANFA	transketolase
251	Creatine kinase B-type OS=Canis familiaris GN=CKB PE=1 SV=1	KCRB_CANFA	Creatine kinase B
252	Uncharacterized protein (Fragment) OS=Canis familiaris GN=ARSA PE=4 SV=1	F6PKZ1_CANFA (+1)	Arylsulfatase A
253	Haptoglobin OS=Canis familiaris GN=HP PE=3 SV=1	G1K2D9_CANFA (+1)	Haptoglobin
254	Uncharacterized protein OS=Canis familiaris GN=ATP6V1A PE=3 SV=1	E2QYG6_CANFA	ATPase, H+ Transporting, Lysosomal 70kDa, V1 Subunit A

Supplementary Table 1 contd.

S. No.	Identified Proteins	Accession Number	Protein Name-FASTA
255	Uncharacterized protein OS=Canis familiaris GN=ARHGDIA PE=4 SV=2	F1PL93_CANFA	Rho GDP Dissociation Inhibitor (GDI) Alpha
256	Proteasome subunit beta type OS=Canis familiaris GN=PSMB6 PE=3 SV=1	E2R0B6_CANFA	Proteasome subunit beta
257	Uncharacterized protein (Fragment) OS=Canis familiaris GN=ITIH5 PE=4 SV=2	F1PDN8_CANFA (+1)	Inter-Alpha-Trypsin Inhibitor Heavy Chain Family, Member 5
258	Uncharacterized protein OS=Canis familiaris GN=IMPA1 PE=4 SV=1	J9P7W8_CANFA	Inositol(Myo)-1(Or 4)-Monophosphatase 1
259	Uncharacterized protein (Fragment) OS=Canis familiaris GN=SERPING1 PE=3 SV=2	F1PYX9_CANFA	Serpin Peptidase Inhibitor, Clade G (C1 Inhibitor), Member 1
260	Uncharacterized protein OS=Canis familiaris GN=ENPP2 PE=4 SV=1	F6UVK4_CANFA	Ectonucleotide Pyrophosphatase/Phosphodiesterase Family Member 2,Extracellular Lysophospholipase D
261	Uncharacterized protein OS=Canis familiaris GN=SERPINA3 PE=3 SV=2	F1PH86_CANFA (+1)	Serine (Or Cysteine) Proteinase Inhibitor, Clade A (Alpha-1Antiproteinase, Antitrypsin), Member 31
262	Uncharacterized protein OS=Canis familiaris GN=SERPINE2 PE=3 SV=1	F6Y2H4_CANFA	Serine (Or Cysteine) Proteinase Inhibitor, Clade E (Nexin
263	Uncharacterized protein OS=Canis familiaris GN=SLC44A1 PE=4 SV=2	E2R949_CANFA	Solute Carrier Family 44 (Choline Transporter), Member 1
264	Uncharacterized protein OS=Canis familiaris GN=ABHD14B PE=4 SV=1	E2RLZ9_CANFA	Abhydrolase Domain Containing 14B
265	Uncharacterized protein OS=Canis familiaris GN=PEPD PE=3 SV=2	F1PNS6_CANFA	Peptidase D
266	Keratin, type II cytoskeletal 2 epidermal OS=Canis familiaris GN=KRT2 PE=3 SV=2	F1PTX4_CANFA (+1)	Keratin, type II cytoskeletal 2
267	Uncharacterized protein OS=Canis familiaris GN=KRT72 PE=3 SV=2	E2QUT7_CANFA	KRT72
268	Uncharacterized protein OS=Canis familiaris GN=KRT8 PE=3 SV=2	F1PW98_CANFA	KRT8
269	Keratin, type II cytoskeletal 1 OS=Canis familiaris GN=KRT1 PE=3 SV=1	F1PTY1_CANFA (+1)	KRT1
270	Uncharacterized protein (Fragment) OS=Canis familiaris GN=KRT5 PE=3 SV=1	L7N095_CANFA	KRT5
271	Uncharacterized protein OS=Canis familiaris GN=KRT3 PE=3 SV=1	L7N094_CANFA	KRT3
272	Uncharacterized protein OS=Canis familiaris GN=KRT5 PE=3 SV=2	E2R8Z5_CANFA	KRT5
273	Uncharacterized protein OS=Canis familiaris GN=KRT74 PE=3 SV=1	L7N0P4_CANFA	KRT74
274	Uncharacterized protein (Fragment) OS=Canis familiaris GN=KRT7 PE=3 SV=2	F1PRB0_CANFA	KRT7
275	Cluster of Uncharacterized protein OS=Canis familiaris GN=KRT19 PE=3 SV=1 (F1Q0N9_CANFA)	F1Q0N9_CANFA [6]	KRT19
276	Uncharacterized protein OS=Canis familiaris GN=KRT19 PE=3 SV=1	F1Q0N9_CANFA	KRT19
277	Keratin, type I cytoskeletal 10 OS=Canis familiaris GN=KRT10 PE=3 SV=2	F1PYU9_CANFA (+1)	KRT10
278	Uncharacterized protein OS=Canis familiaris GN=KRT42 PE=3 SV=1	E2R7W6_CANFA	KRT42
279	Uncharacterized protein OS=Canis familiaris GN=KRT14 PE=3 SV=2	F1Q0R0_CANFA	KRT14
280	Uncharacterized protein OS=Canis familiaris GN=KRT17 PE=3 SV=2	F1Q0Q9_CANFA	KRT17
281	Cluster of Uncharacterized protein (Fragment) OS=Canis familiaris GN=KRT18 PE=3 SV=2 (E2REU6_CANFA)	E2REU6_CANFA [2]	KRT18
282	Uncharacterized protein (Fragment) OS=Canis familiaris GN=KRT18 PE=3 SV=2	E2REU6_CANFA	KRT18
283	Uncharacterized protein OS=Canis familiaris GN=KRT32 PE=3 SV=1	L7N0G9_CANFA	KRT32
284	Keratin, type I cytoskeletal 9 OS=Canis familiaris GN=KRT9 PE=4 SV=1	F1Q0N7_CANFA (+3)	KRT9
285	Uncharacterized protein OS=Canis familiaris GN=KRT82 PE=3 SV=1	E2R831_CANFA	KRT82
286	Uncharacterized protein (Fragment) OS=Canis familiaris PE=4 SV=2	F1PQI2_CANFA	
287	Uncharacterized protein OS=Canis familiaris PE=4 SV=2	F1PCM6_CANFA	
288	Uncharacterized protein OS=Canis familiaris GN=LOC100856711 PE=4 SV=1	J9P1I7_CANFA (+1)	LOC100856711

Supplementary Table S1 contd.

S. No.	Identified Proteins	Accession Number	Protein Name-FASTA
**289**	Uncharacterized protein OS=Canis familiaris PE=3 SV=1	J9NTU0_CANFA	
**290**	Uncharacterized protein OS=Canis familiaris PE=3 SV=2	F1PCH3_CANFA	
**291**	Uncharacterized protein OS=Canis familiaris PE=4 SV=1	F6Y478_CANFA	
**292**	Cluster of Uncharacterized protein OS=Canis familiaris PE=4 SV=2 (E2RCC8_CANFA)	E2RCC8_CANFA	
**293**	Uncharacterized protein OS=Canis familiaris PE=4 SV=2	E2RCC8_CANFA	
**294**	Uncharacterized protein (Fragment) OS=Canis familiaris PE=3 SV=1	J9P219_CANFA	
**295**	Uncharacterized protein OS=Canis familiaris PE=4 SV=1	J9P9V0_CANFA	
**296**	Cluster of Uncharacterized protein OS=Canis familiaris GN=LOC100687814 PE=3 SV=1 (J9NW97_CANFA)	J9NW97_CANFA [4]	LOC100687814
**297**	Uncharacterized protein OS=Canis familiaris GN=LOC100687814 PE=3 SV=1	J9NW97_CANFA	LOC100687814
**298**	Uncharacterized protein OS=Canis familiaris GN=LOC100856491 PE=4 SV=1	J9P889_CANFA	LOC100856491
**299**	Uncharacterized protein OS=Canis familiaris GN=LOC612122 PE=4 SV=2	H9GWR0_CANFA (+4)	LOC612122
**300**	Uncharacterized protein OS=Canis familiaris GN=LOC609095 PE=3 SV=1	E2RQN4_CANFA	LOC609095
**301**	Uncharacterized protein (Fragment) OS=Canis familiaris GN=LOC609010 PE=4 SV=1	J9P0V2_CANFA	LOC609010
**302**	Uncharacterized protein (Fragment) OS=Canis familiaris GN=LOC609402 PE=3 SV=2	E2RLH6_CANFA (+1)	LOC609402
**303**	Uncharacterized protein (Fragment) OS=Canis familiaris GN=LOC100856547 PE=4 SV=2	H9GWE7_CANFA (+2)	LOC100856547
